# Different stress responsive strategies to drought and heat in two durum wheat cultivars with contrasting water use efficiency

**DOI:** 10.1186/1471-2164-14-821

**Published:** 2013-11-22

**Authors:** Alessio Aprile, Lenka Havlickova, Riccardo Panna, Caterina Marè, Grazia M Borrelli, Daniela Marone, Carla Perrotta, Patrizia Rampino, Luigi De Bellis, Vladislav Curn, Anna M Mastrangelo, Fulvia Rizza, Luigi Cattivelli

**Affiliations:** Department of Biological and Environmental Sciences and Technologies, University of Salento, Prov.le Lecce Monteroni, I-73100 Lecce, Italy; Consiglio per la Ricerca e la Sperimentazione in Agricoltura, Genomics Research Centre, via San Protaso 302, I-29107 Fiorenzuola d’Arda (PC), Italy; Biotechnological Centre, Faculty of Agriculture, University of South Bohemia, CZ-370 05 České Budějovice, Czech Republic; Consiglio per la Ricerca e la Sperimentazione in Agricoltura, Cereal Research Centre, SS16 km 675, 71122 Foggia, Italy

## Abstract

**Background:**

Durum wheat often faces water scarcity and high temperatures, two events that usually occur simultaneously in the fields. Here we report on the stress responsive strategy of two durum wheat cultivars, characterized by different water use efficiency, subjected to drought, heat and a combination of both stresses.

**Results:**

The cv Ofanto (lower water use efficiency) activated a large set of well-known drought-related genes after drought treatment, while Cappelli (higher water use efficiency) showed the constitutive expression of several genes induced by drought in Ofanto and a modulation of a limited number of genes in response to stress. At molecular level the two cvs differed for the activation of molecular messengers, genes involved in the regulation of chromatin condensation, nuclear speckles and stomatal closure. Noteworthy, the heat response in Cappelli involved also the up-regulation of genes belonging to fatty acid β-oxidation pathway, glyoxylate cycle and senescence, suggesting an early activation of senescence in this cv. A gene of unknown function having the greatest expression difference between the two cultivars was selected and used for expression QTL analysis, the corresponding QTL was mapped on chromosome 6B.

**Conclusion:**

Ofanto and Cappelli are characterized by two opposite stress-responsive strategies. In Ofanto the combination of drought and heat stress led to an increased number of modulated genes, exceeding the simple cumulative effects of the two single stresses, whereas in Cappelli the same treatment triggered a number of differentially expressed genes lower than those altered in response to heat stress alone. This work provides clear evidences that the genetic system based on Cappelli and Ofanto represents an ideal tool for the genetic dissection of the molecular response to drought and other abiotic stresses.

**Electronic supplementary material:**

The online version of this article (doi:10.1186/1471-2164-14-821) contains supplementary material, which is available to authorized users.

## Background

Heat and drought stress and their combination are the most important stresses experienced by plants and they are responsible of a large fraction of productivity losses [1]. Plants respond to stress with a wide range of modifications leading to changes at morphological, cellular, physiological, biochemical, and molecular level [2, 3]. A relevant component of the plant adaptation to stress conditions is dependent on transcriptional changes and the expression of key genes results in enhanced stress tolerance [4, 5]. Overall, the molecular response of plants to abiotic stress is mediated by a number of molecules involved in signal transduction leading to the activation of specific gene networks resulting from the re-programming of cell expression machinery. To these networks belong genes coding for a variety of proteins involved in DNA remodeling, transcription regulation, protein modifications, etc. [6]. A number of publications described the transcriptional changes induced in response to drought [7–9] and heat [10, 11] stresses, however much less is known when plants are simultaneously subjected to drought and heat stress, an event very common under field conditions. Several works indicate that the molecular response to the combination of heat and drought activates networks that are different from those activated by heat or drought stress taken singularly [12–15]. Furthermore, most of the publications cited above have been carried out on seedlings and therefore might not reflect exactly the molecular response of crops exposed to stress when plants are in more advanced growing stages.

Durum wheat is an important cereal crop grown mainly in semi-arid environments (e.g. Mediterranean regions) characterized by water scarcity and high temperatures often occurring at the same time. The two durum wheat cultivars Cappelli and Ofanto, contrasting for many agronomic and physiological traits, have been extensively characterized [16–18]. Measures based on stomata conductance and on grain carbon isotope discrimination from field trials and growth chamber experiments consistently showed a higher water use efficiency (WUE) in Cappelli compared to Ofanto, a finding correlated with a different stomata conductance (lower in Cappelli) over a range of relative soil water contents [17]. A RIL segregating population with a corresponding molecular marker map has also been developed from the cross between Ofanto and Cappelli [19, 20] and used to localize QTLs for leaf porosity and chlorophyll content in field conditions [18].

This work reports on a microarray-based transcriptomic analysis carried out on the durum wheat cultivars Cappelli and Ofanto grown to booting stage and subjected to heat, drought and to a combination of drought and heat stresses, conditions similar to the experience of a crop grown in Mediterranean environments and exposed to a terminal heat/drought stress. Furthermore, several selected drought-related genes have been tested in the same cultivars exposed to drought at tillering stage to confirm the constitutive nature of the different stress response strategy detected at booting stage. A gene selected among those characterized by different stress response between the two cultivars was used for an expression QTL analysis and the corresponding QTL was mapped on chromosome 6B.

## Results and discussion

### Sample preparation and hybridization quality

Plants of two *Triticum turgidum* subsp*. durum* cultivars, Ofanto and Cappelli, were grown in controlled conditions and exposed to drought stress (DS), heat stress (HS) and to a combination of heat and drought stresses (CS) when they reached the booting stage. Control (Ctrl) plants were watered to maintain a Soil Water Content (SWC) equal to 28%. DS was imposed by withholding water and allowing the pots to reach a SWC equal to 12.5%, while HS was imposed progressively exposing the plants for two days at 30°C followed by additional two days at 34°C, and subsequently to 40°C. For CS the two treatments were combined in order to achieve simultaneously 12.5% SWC and 40°C. More details are reported in the Methods section. Table 1 reports the flag leaf temperature and the Relative Water Content (RWC%) determined on the same plants used for RNA isolation immediately before sampling. Cappelli was characterized by higher leaf temperature compared to Ofanto, a finding in agreement with a lower transpiration capacity due to a lower stomatal conductance as reported by Rizza et al. [17]. Nevertheless, Cappelli showed also a lower RWC compared to Ofanto, a condition that could be explained by a different capacity of water uptake from soil.

**Table 1:** **Relative Water Content (RWC) and leaf temperature of durum wheat plants subjected to drought, heat and to a combination of drought and heat stress**

Genotype	Stress condition	RWC (%)	Leaf temperature (°C)
**Ofanto**	Control	96.97 ± 1.47	21.62 ± 0.16
	Drought stress	94.04 ± 1.08	21.70 ± 0.07
	Heat stress	85.96 ± 2.02	35.42 ± 0.65
	Combined stress	59.34 ± 2.34	38.36 ± 0.98
**Cappelli**	Control	95.86 ± 1.06	22.50 ± 0.55
	Drought stress	88.68 ± 0.54	22.00 ± 0.10
	Heat stress	82.88 ± 0.45	36.50 ± 0.50
	Combined stress	38.53 ± 8.02	41.00 ± 0.20

RWC and temperature were determined on the leaf below flag leaf that was used for transcriptomic analysis.

Total RNA was isolated from the flag leaves and used for microarray hybridization.

The comparison of the “RNA degradation plots” graphs obtained in this experiment (Additional file 1) with those of 18 experiments stored in the PlexDB database from 2005 to 2013 (http://www.plexdb.org) [21] supports the high quality of the hybridizations reported in this work. The observed average background ranged from 37.4 to 46.0 units of expression, well within the parameters defined by Affymetrix, and comparable with the values obtained in other experiments that have used the GeneChip^®^ Wheat Genome Array [7, 22]. The “present call” percentage ranged between 36.7% and 42.8% among the 61,000 probe sets indicating a degree of variability among the analyzed samples. The Pearson correlation was calculated between pairs of replications within each sample. The values ranged from 0.922 to 0.998.

Four probe sets, representing genes putatively involved in the fatty acid metabolism (listed in Methods) were subjected to real-time qRT-PCR analysis to validate the microarray data. The observed Pearson correlation between microarray and qRT-PCR data was 0.753 (Additional file 2).

### Identification of differentially expressed genes

**Figure 1:**
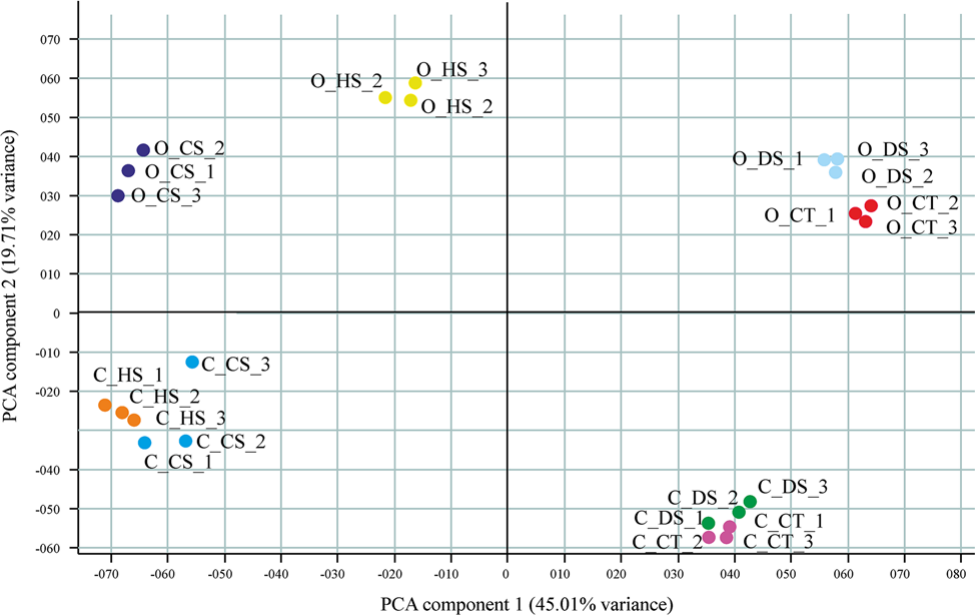
**Principal Component Analysis (PCA plot) of the wheat array hybridization data.** The x and y axis represent the two principal components of the total variance, 45.01% and 19.71%, respectively. Each dot represents a single repetition of Ofanto and Cappelli RNA samples subjected to different stress conditions. C_CT and O_CT = Cappelli control and Ofanto control samples; C_DS and O_DS = Drought stressed samples; C_HS and O_HS = Heat stressed samples; C_CS and O_CS = Combined stress samples.

The PCA (Principal Component Analysis) identified two principal components explaining 45.0% and 19.7% of the observed variability (Figure 1). The first component (x-axis) accounts for the treatment effect, while along the second component (y-axis), the samples are separated according to the genotype. The vicinity between control samples (Ctrl) and drought stressed samples (DS) suggests a smaller effect of drought treatment compared to heat stress (HS) and to the combination of heat and drought stress (CS).

**Figure 2:**
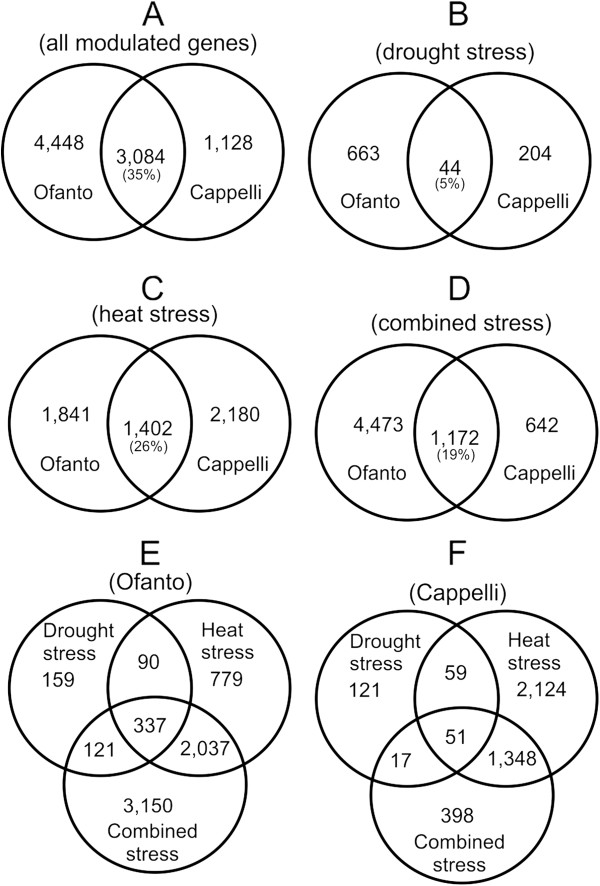
**Venn diagrams representing the distribution of the genes in the different classes. A**: All genes modulated in the experiment (drought stress + heat stress + combined stress) are separated according to the tested genotype. **B**: All genes modulated in response to drought stress are separated according to the tested genotype. **C**: All genes modulated in response to heat stress are separated according to the tested genotype. **D**: All genes modulated in response to combined stress (drought + heat stress) are separated according to the tested genotype. **E**: All genes modulated in response to stress in the durum wheat cultivar Ofanto are separated according to the stress conditions. **F**: All genes modulated in response to stress in the durum wheat cultivar Cappelli are separated according to the stress conditions.

To identify the genes modulated in response to the applied stress conditions, five comparisons were carried out for each cultivar: Ctrl *vs* DS, Ctrl *vs* HS, Ctrl *vs* CS, DS *vs* CS and HS *vs* CS. Differentially expressed genes were identified based on Welch t-test with Benjamini and Hochberg correction for multiple testing [23]. A gene was considered differentially expressed when the *p-*value was less than 0.01 and the ratio of induction or repression was equal to or greater than 2. The analysis yielded 4,212 not redundant differentially expressed probe sets (PSs) in Cappelli and 7,532 in Ofanto. Among these, 3,084 PSs differentially expressed were in common to both genotypes (Figure 2A). DS led to the modulation of 707 PSs in Ofanto and 248 in Cappelli with only 44 (27 up- and 17 down-regulated) in common (Figure 2B), while HS triggered the modulation of 3,243 PSs in Ofanto and 3,582 PSs in Cappelli with 1,402 PSs in common (Figure 2C). Moreover, a combination of drought and heat stress activated or repressed 5,645 PSs in Ofanto, and only 1,814 in Cappelli with 1,172 sequences in common (Figure 2D). Although the different number of PSs modulated in response to DS and HS can be due to different stress intensity (a more severe DS might lead to a higher number of differentially expressed genes), these results highlight very clearly that Cappelli and Ofanto have different stress response strategies with a minimal overlapping in terms of genes modulated in response to identical stress conditions. This finding was more evident in response to drought both as drought alone (DS) or in combination with heat (CS). In both conditions Ofanto modulated the expression of about three times more PSs than Cappelli.

The analysis of the 525 PSs up-regulated in response to DS in Ofanto and of their annotations highlighted that many of them were characterized by well-known drought-related functions. Furthermore, functional categories related to osmotic, salt, heat and cold response were over-represented among the annotation list. For instance dehydrins (*At1g01470, LEA14*, [Ta.25026.1.S1_at]*; At1g47128, RD21A*, [Ta.5812.1.A1_at]*; At4g39090, RD19A*, [Ta.4419.2.S1_x_at]*; At5g25610, RD22*, [Ta.28209.3.S1_at, Ta.28209.3.S1_x_at]*; At2g38905, Low temperature and salt response protein*, [Ta.27725.1.S1_at]*; At3g50970, LTI30/Xero2,* [TaAffx.131747.1.S1_x_at]*; At4g36720, HvA22*, [Ta.3038.2.S1_a_at]*; At5g66400, RAB18,* [10 different PSs, see Additional file 3]), lipid transfer proteins (*At5g59310, LTP4*, [Ta.1337.1.S1_x_at]*; At5g59320, LTP3*, [Ta.28368.2.S1_at]), heat shock proteins (*At1g74310, HSP101*, [TaAffx.56160.1.S1_at]*; At3g09440, HSP70*, [Ta.23807.1.S1_x_at]*; At5g49910, CPHSC70-2*, [TaAffx.114770.1.S1_at]), stress-related transcription factors (*At4g38620, MYB4*, [Ta.26049.1.S1_a_at]*; At2g46270, GBF3*, [Ta.13357.2.S1_at]), ABA and drought responsive proteins (*At1g35720, ANN1*, [Ta.14590.1.S1_a_at]*; At5g03280, EIN2*, [Ta.7910.1.A1_at]*; At2g26980, CIPK3*, [TaAffx.106682.1.S1_at]) and genes coding proteins involved in drought responsive proline biosynthesis (*At3g55610, P5CS*, [Ta.7091.1.S1_at]*; At5g14800, P5CR*, [Ta.591.1.S1_at]) were all up-regulated by DS in Ofanto (Additional file 3). The identification of well-known drought-related genes in Ofanto plants exposed to DS demonstrates that the stress conditions applied were effective in triggering a typical drought response in Ofanto, but not in Cappelli. This finding suggests, once again, a different perception of drought stress and, consequently, a different molecular response of the two cultivars.

Notably, 7 PSs corresponding to several genes (*LEA14*, *ANN1*, *RD21A*, *HSP101*, *HSP70*, *CPHSP70-2*, *LTP4*) induced by drought stress in Ofanto but not in Cappelli, showed a higher expression level in Cappelli Ctrl *vs* Ofanto Ctrl. These data suggest that a part of the genes expressed in Ofanto in response to drought were constitutively expressed in Cappelli. On the contrary, none of the 651 PSs expressed at higher level in Ofanto Ctrl *vs* Cappelli Ctrl are known to be related to stress response.

In the cultivar Ofanto, CS treatment led to the modulation of about 5,645 PSs. Of these, 2,374 were in common with HS, 558 PSs were shared with the DS response and 3,150 PSs were specific of the combined treatment (Figure 2E). In Cappelli, CS modulated 1,814 PSs of which 68 were also modulated in response to DS and 1,399 in response to HS (Figure 2F). These results confirm previous findings [12–15] and highlight that the effect of CS is not a simple sum of drought and heat stress, rather it is perceived as a new stress condition.

### QT-clustering analysis identifies different expression profiles in Cappelli and Ofanto

Besides the genes commonly regulated in the two cultivars, the stress response of Ofanto and Cappelli was characterized by significant differences that might be associated to the different physiological behavior of these genotypes under drought stress [17]. To highlight commonalities and differences between the two cultivars, a QT-cluster analysis was performed to identify groups of genes with an expression profile associated to specific or common responses in the tested genotypes. The QT-clustering algorithm (Pearson correlation higher than 0.8 and minimum cluster size of 30 probe sets), run with 8,660 differentially expressed PSs, identified 52 clusters grouping 5,910 PSs plus 2,750 unclassified PSs (Additional file 4). Visual analysis of the 52 clusters highlighted a wide range of expression behaviors, no gene cluster was characterized by opposite trends between Ofanto and Cappelli (i.e. up-regulated in one cultivar and down-regulated in the other one). Some clusters showed an almost identical expression profile in the two cultivars (e.g. clusters 5, 13, 14, 21, 35 and 37 with 649 PSs in total), while the others highlighted some differences in gene expression between Ofanto and Cappelli.

Looking at the whole transcriptome numbers (Figure 2E and 2F) the most evident difference between the stress response of Ofanto and Cappelli was the much stronger response of Cappelli to heat stress. In this genotype 85% of all PSs modulated in the experiment were responsive to HS (3,582 PSs out of 4,218; in Ofanto 49%). Furthermore, the PSs regulated by HS and CS in Cappelli represent 77% of all PSs regulated in response to CS in this genotype (in Ofanto 36%).

**Figure 3:**
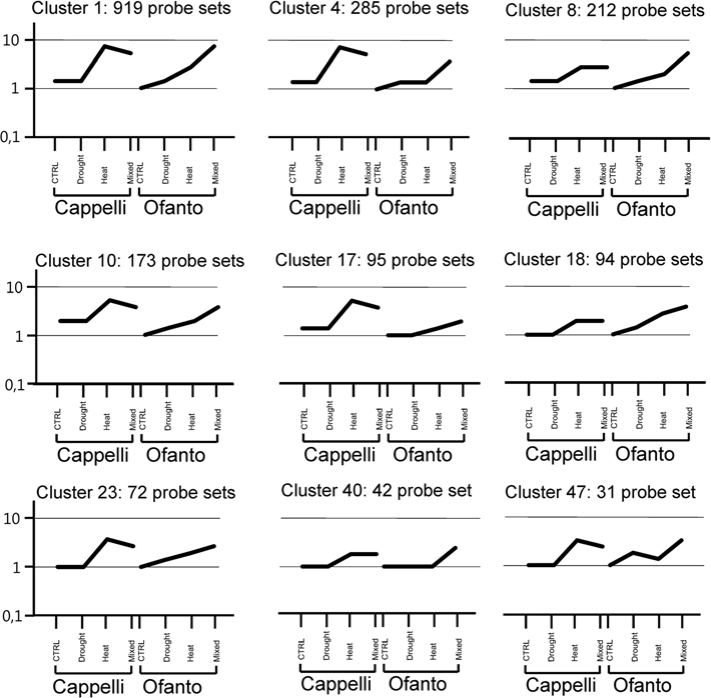
**QT-clustering analysis obtained using the expression values of the 8,660 stress-related genes differentially expressed in at least one condition/genotype.** The analysis was performed with a minimum cluster size of 30 and a correlation value of 0.80. The four treatment conditions, grouped by genotypes, are plotted on x axis. The relative expression level (for each probe set the data were normalized to the median expression level of the Ofanto Ctrl samples) is plotted on the y axis. The lines represent the mean expression trend of all probe sets belonging to each cluster. The nine clusters (cluster 1, 4, 8, 10, 17, 18, 23, 40 and 47) contain genes whose expression might be associated to the different molecular response of Ofanto and Cappelli (details in the Results). The whole data set of 52 clusters is presented in the Additional file 4.

Some clusters highlighted PSs with a contrasting expression trend in the two cultivars across all treatments considered, these clusters could contain genes whose expression might contribute to explain the different molecular response of Ofanto and Cappelli to drought and heat stress. In this context the most relevant are the 9 clusters (namely clusters 1, 4, 8, 10, 17, 18, 23, 40 and 47) showed in Figure 3 and representing a total of 1,923 PSs, all up-regulated. In Cappelli, these genes were characterized by no or almost no response to DS, while, their expression in response to HS and CS was definitely enhanced. In Ofanto, the same clusters were up-regulated in at least one comparison among Ctrl *vs* DS, Ctrl *vs* HS, Ctrl *vs* CS. Overall the expression level of these genes was maximum under HS in Cappelli, while in Ofanto it was more elevated in response to CS. Since these clusters represent different gene regulation mechanisms, a specific data mining work has been undertaken to identify the key functions and pathways underlined by the selected clusters.

### Signaling components

Among the 1,923 PSs belonging to clusters 1, 4, 8, 10, 17, 18, 23, 40 and 47 there were many components with a known role in the molecular events leading to stress tolerance (Table 2).

**Table 2:** **Overview of the signalling component genes up-regulated by drought, heat and combined stress and belonging to cluster 1, 4, 8, 10, 17, 18, 23, 40 and 47**

Signaling component class	Gene name	AGI code	Probe set ID	Cluster
Ca^2+^ binding protein	*RD20*	At2g33380	Ta.9830.2.S1_at	4
Phosphatidic acid	*PLD*	At3g15730	Ta.11144.1.A1_at	1
		At3g15730	Ta.3908.1.S1_at	1
	*DAG kinase*	At5g07920	Ta.3010.1.A1_at	4
		At2g20900	Ta.3876.1.A1_at	8
Phosphatidylinositol	*PI3K*	At1g60490	Ta.22091.1.A1_at	1
			Ta.28795.1.S1_at	1
	*PI(3,4)K*	At1g13640	TaAffx.110494.1.S1_x_at	1
	*PI4p5K*	At4g33240	Ta.5574.1.S1_a_at	23
			TaAffx.86994.1.S1_at	1
		At3g14270	Ta.5574.3.S1_at	1
	*AtVPS15*	At4g29380	Ta.8384.1.S1_at	8
Chromatin condensation	*Histone 4*	At5g59970	Ta.10329.23.S1_at	1
			Ta.10329.8.S1_x_at	1
	*Histone 1.2*	At2g30620	Ta.23485.1.S1_at	1
	*Histone 2A*	At4g27230	Ta.644.1.S1_at	1
		At1g54690	Ta.6538.2.S1_at	4
	*Histone 2B*	At2g37470	Ta.7378.28.S1_x_at	4
	*Histone 3*	At5g10400	Ta.27101.7_S1_at	40
		At5g10400	Ta.27101.7_S1__xat	40
	*HDAC*	At3g44680	TaAffx.93321.1.S1_at	1
		At5g61060	Ta.1597.1.S1_at	1
	*HAT*	At5g64610	Ta.5414.1.S1_at	1
			Ta.5414.2.S1_a_at	8
	*RCC1*	At3g02300	Ta.15822.1.S1_at	1
		At5g48330	TaAffx.53414.1.S1_at	8
	*SMC2-like condensin*	At5g62410	Ta.20443.1.A1_at	17
	*AtMSI1*	At5g58230	Ta.13849.1.S1_at	1
Stomatal closure	*OST1*	At4g33950	Ta.991.1.S1_a_at	1
			Ta.991.1.S1_at	1
			Ta.991.1.S1_x_at	1
			Ta.2551.1.S1_at	4
			Ta.6040.1.S1_a_at	18
Temperature signaling	*CHY*	At5g65940	Ta.6750.2.S1_a_at	1
			Ta.6750.3.S1_a_at	1
			Ta.6750.3.S1_x_at	1
			TaAffx.108337.1.S1_s_at	1
	*BCKDH*	At3g06850	Ta.12252.1.S1_s_at	4
			Ta.8293.1.A1_at	4
	*HSF*	At3g22830	Ta.28772.1.S1_at	1
		At2g26150	TaAffx.105519.1.S1_at	4
	*HSPs*	45 different probe sets ID		

An early response to stress is represented by the activation of trans-membrane proteins acting as calcium transport channels resulting in a Ca^2+^ flow into the cytosolic region [24]. In *Arabidopsis* the calcium-binding RD20 protein (*At2g33380*) plays a key role in drought tolerance through stomatal control under water deficit condition [25]. On the GeneChip^®^ Wheat Genome Array six PSs with a high sequence similarity to the *RD20* gene are present, among them, one (Ta.9830.2.S1_at) was modulated by stress in the experiment here reported and exhibits an expression profile belonging to cluster 4 (Additional file 5). Its transcription was strongly activated in Cappelli in response to HS and CS, while in Ofanto it was activated only by CS (Additional file 5). Besides calcium binding activity, RD20 can play a role as peroxygenase in the oxylipin pathway [26]. Oxylipins are fatty acid hydroperoxide compounds active as germination inhibitors of fungal spores [27] and as modulators of plant development processes [28]. Nevertheless, when we analyzed the expression of all genes involved in the oxylipin pathway, no differentially expressed genes in response to stress were found, suggesting that the up-regulation of *RD20*, in our experiment, is not linked to oxylipin pathway regulation. Several other genes coding for Ca^2+^ binding proteins but without EF-hand motifs, like phospholipase D (PLD) were also found. The PLD catalyzes the cleavage of membrane phospholipids into phosphatidic acid (PA) and a soluble head group; its activity is regulated by [Ca^2+^]. PA, in turn, is a second messenger implicated in plant stress signaling [6], also produced by diacyl-glycerol kinase (DAG kinase) [29]. Two PSs (Ta.11144.1.A1_at, Ta.3908.1.S1_at) with high sequence similarity to *Arabidopsis PLD* (*At3g15730*) were differentially expressed with an expression profile following the one of cluster 1. Moreover two *DAG kinase* (*At5g07920*, *At2g20900*) related probe sets (Ta.3010.1.A1_at, Ta.3876.1.A1_at) belonging to clusters 4 and 8 (Additional file 5) were also differentially expressed.

Another key messenger in plants is the phosphatidylinositol (PI) that can be phosphorylated at several positions of the inositol head group. In the experiment here described, *PI3K* (*At1g60490* [Ta.22091.1.A1_at, Ta.28795.1.S1_at]), *PI(3,4)K* (*At1g13640* [TaAffx.110494.1.S1_x_at]) and *PI4p5K* (*At4g33240* [Ta.5574.1.S1_a_at, TaAffx.86994.1.S1_at]; *At3g14270* [Ta.5574.3.S1_at]) were differentially expressed and exhibited the same expression trend of cluster 1, but for *PI(3,4)K* belonging to cluster 23. Notably, it has been reported that PI3K is required for ABA-induced ROS generation that leads to stomatal closure in *Vicia faba*[30]. Overall these data suggest that the calcium-related messengers and phosphoinositide signaling are involved in the stress response and that they are more active in Cappelli compared to Ofanto when exposed to HS (see the expression profiles of clusters 1 and 4, Figure 3).

An increased expression of genes encoding proteins involved in epigenetic regulation has also been associated to stress response. The variations of the chromatin structure are often dependent on the expression of histone variants or on histone post-translational modifications [31]. The drought, heat and combined stress activate the expression of different histone variants such as histone 4 (*At5g59970* [Ta.10329.23.S1_at, Ta.10329.8.S1_x_at]), histone 1.2 (*At2g30620* [Ta.23485.1.S1_at]), histone 2A (*At4g27230* [Ta.644.1.S1_at], *At1g54690* [Ta.6538.2.S1_at]), histone 2B (*At2g37470* [Ta.7378.28.S1_x_at]), histone 3 (*At5g10400* [Ta.27101.7.S1_at, Ta.27101.7.S1_x_at]), two histone deacetylases (*At3g44680* [TaAffx.93321.1.S1_at], *At5g61060* [Ta.1597.1.S1_at]) and a histone acetyltransferase (*At5g64610* [Ta.5414.1.S1_at, Ta.5414.2.S1_a_at]), whose expression behaviors were associated mainly to cluster 1, and to some extent also to clusters 4, 8 and 40. Moreover two regulators of chromosome condensation, *RCC1* (*At3g02300* [Ta.15822.1.S1_at], *At5g48330* [TaAffx.53414.1.S1_at], cluster 1 and 8, respectively) and a *SMC2-like condensin* (*At5g62410* [Ta.20443.1.A1_at] cluster 17), were differentially expressed. Besides regulator of chromosome condensation genes, the WD-40 repeat protein (*At5g58230* [Ta.13849.1.S1_at]), also named *At*MSI1, was described to be involved in chromatin assembly [32].The *msi1 Arabidopsis* mutant exhibits an enhanced expression of many ABA-responsive genes eliciting the response to drought and salt stress. Alexandre *et al*. [32] have demonstrated that MSI1 can bind to the chromatin of the drought-inducible downstream target *RD20* suggesting for MSI1 a role in the negative regulation of *Arabidopsis* drought-stress response. However, in the durum wheat transcriptome analysis here reported, the up-regulation of *AtMSI1* was directly correlated to the expression of ABA-responsive genes and *RD20*. Fourteen probe sets with high sequence similarity to the gene coding the WD-40 repeat protein (clusters 1, 4, 8, 10, 17, 18, 23, 40 and 47) were found also by the present analysis (Additional file 5). Many of them are annotated as components of CUL4-based E3 ubiquitin ligases [33]. In particular *At4g29380* [Ta.8384.1.S1_at] (also named *AtVPS15*) is a component of CUL4-based E3 ubiquitin ligases, but has a PI3K activity too, and is directly involved in PI3P production [34].

The analysis of the gene clusters differentially expressed between Ofanto and Cappelli during stress response identified also 21 probe sets related to proteins containing the RNA recognition motif (RRM) and splicing factors. These proteins were reported to be associated to the mRNA metabolism of genes involved in the ABA signal transduction in *Arabidopsis*[35] as well as in *Vicia faba*[36, 37]. One of these proteins (AKIP1) is constitutively expressed in the nucleus of stomata guard cells, and it aggregates in small structures (speckles) involved in mRNA storage or processing, in response to ABA. Moreover AKIP1 is activated through phosphorylation by AAPK (ABA Activated Protein Kinase) and, once activated, is able for example to bind dehydrin mRNAs [37]. In *Arabidopsis* the *AAPK* homolog gene (protein kinase OPEN stomata 1, *OST1*, *At4g33950*, also known as *SnRK2E* or *SnRK2.6*) is up-regulated by ABA and plays a key role in the phosphorylation events that lead to the ABA and osmotic induced stomata closure [38–41]. The wheat GeneChip^®^ carries nine probe sets that have a sequence similarity with *OST1*. Of these, five were found to be up-regulated in durum wheat with an expression profile belonging to cluster 1 (Ta.991.1.S1_a_at, Ta.991.1.S1_at, and Ta.991.1.S1_x_at), cluster 4 (Ta.2551.1.S1_at) and cluster 18 (Ta.6040.1.S1_a_at). Another probe set, Ta.6918.1.S1_at, was identified in cluster 21 (Additional file 3).

*CHY* is a gene (*At5g65940*) coding for a β-hydroxyisobutyryl CoA hydrolase (EC 3.1.2.4) involved in the catabolism of valine and with a role in the perception and transduction of low temperature signaling [42]. In durum wheat four probe sets (Ta.6750.2.S1_a_at, Ta.6750.3.S1_a_at, Ta.6750.3.S1_x_at, TaAffx.108337.1.S1_s_at) with high sequence similarity to the *CHY* were found in cluster 1. A second gene involved in valine pathway and signal transduction was also differentially expressed; the branched chain alpha-keto acid dehydrogenase (BCKDH, EC 1.2.1.25) is localized in the mitochondria and catalyzes the reaction that leads to the synthesis of isobutyryl-CoA, a substrate of the reaction leading to hydroxyl-isobutyrate, catalyzed by CHY [43]. The wheat GeneChip^®^ carries three probe sets with sequence similarity to *BCKDH*, two of them (Ta.12252.1.S1_s_at, Ta.8293.1.A1_at) were up-regulated according to cluster 4 expression profile.

The more pronounced response to HS of Cappelli *vs* Ofanto was also supported by two heat-shock factors (*HSF*s, [44]) related probe sets found in clusters 1 and 4, (*At3g22830*, Ta.28772.1.S1_at and *At2g26150*, TaAffx.105519.1.S1_at, respectively) whose expression promoted the up-regulation of 44 *HSP-*related probe sets exhibiting a similar expression trend and belonging to clusters 1, 4, 8, 10, 17, 18, 23, 40 and 47.

Overall, the analysis of the genes belonging to clusters characterized by significantly different expression profiles between Cappelli and Ofanto allowed the identification of a number of key components of the heat and drought signaling pathways, suggesting that the different stress response of the two cultivars is supported by a different modulation of the regulatory mechanisms (Table 2).

### The fatty acids β-oxidation pathway

In plants, the β-oxidation takes place mainly in peroxisomes and glyoxysomes. This pathway requires four enzymatic reactions that are repeated until the fatty acid degradation is completed [45]. The first reaction is catalyzed by acyl-CoA ligase/synthase (ACS, EC 6.2.1.3). There are twenty-two probe sets with high sequence similarity to the gene coding for this enzyme on the wheat GeneChip^®^; seven were differentially expressed, among these four have expression trend according to cluster 17.

The activity of the acyl-CoA synthase leads to the formation of acyl-CoA that is converted to trans-2-enoyl-CoA by acyl-CoA oxidase (ACX, EC 1.3.3.6), the true first β-oxidation enzyme [46]. In the array there are 7 probe sets exhibiting sequence similarity to the *Arabidopsis* acyl-CoA oxidase genes (*At5g65110*, *At3g51840*, *At1g06290*), four of which were differentially expressed, three (Ta.10260.1.S1_at, Ta.9335.1.S1_at, Ta.9335.1.S1_s_at) belong to cluster 4, one (Ta.3420.2.S1_a_at) belongs to cluster 10. The previous reaction requires the presence of FAD as co-factor and O_2_ as electron acceptor; the latter is reduced to H_2_O_2_ that is subsequently decomposed by a peroxisome catalase (EC 1.11.1.16). Six peroxisome catalase-related probe sets were differentially expressed, four according to cluster 4 (Ta.1055.1.S1_at, Ta.1055.1.S1_x_at, Ta.1055.2.S1_at, Ta.1055.2.S1_x_at) and two according to cluster 12 (Additional file 3).

Subsequently, the trans-2-enoyl-CoA undergoes a hydration reaction catalyzed by enoyl-CoA hydratase (ECH, EC 4.2.1.17) and it is transformed into the corresponding L-β-hydroxyacyl-CoA; in the next step, catalyzed by the L-β-hydroxyacyl-CoA dehydrogenase (HCD, EC 1.1.1.35), the L-β-hydroxyacyl-CoA is converted into β-ketoacyl-CoA, this transformation requires a molecule of NAD^+^ that is reduced to NADH. In plant peroxisomes a multi-functional protein coded by *At4g29010* is responsible for at least four activities, including the activity of enoyl-CoA hydratase and L-β-hydroxyacyl-CoA dehydrogenase [47]. Two wheat probe sets (Ta.9184.1.S1_at, Ta.2583.1.S1_x_at) have sequence similarity to the *At4g29010* gene and they were both classified in cluster 4.

The ending step of each β-oxidation cycle is the acetyl-CoA detachment from the starting fatty acid, catalyzed by the acyl-CoA-acetyltransferase enzyme or thiolase (AAT, EC 2.3.1.16). One probe set (Ta.28367.1.S1_at) has sequence similarity with the *Arabidopsis* thiolase gene and was induced by stress according to cluster 17.

**Figure 4:**
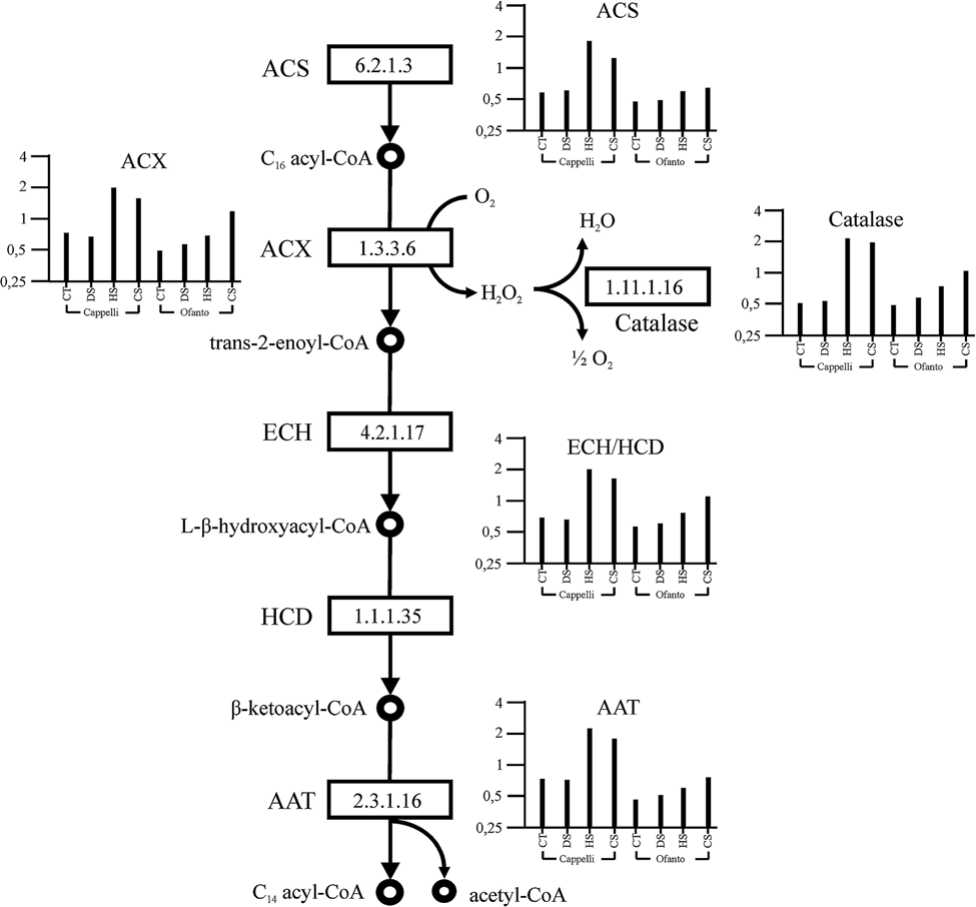
**Representation of the β-oxidation pathway: variations in the expression levels of the genes coding for the key enzymes.** The four treatment conditions, grouped by genotypes, are plotted on x axis. The relative expression level (data normalized to the median for each probe set) is plotted on the y axis. ACS: acyl-CoA synthase (EC 6.2.1.3); ACX: acyl-CoA oxidase (EC 1.3.3.6); Catalase: peroxisome catalase (EC 1.11.1.6); ECH: enoyl-CoA hydratase (EC 4.2.1.17); HCD: L-β-hydroxyacyl-CoA dehydrogenase (EC 1.1.1.35); AAT: acyl-CoA-acetyltransferase enzyme or thiolase (EC 2.3.1.16). CT = Control samples; DS = Drought stressed samples; HS = Heat stressed samples; CS = Combined stress samples.

In summary, all genes involved in the fatty acid β-oxidation, as well as a corollary gene of this cycle (peroxisome catalase), are activated by both HS and CS. The highest level of transcription was achieved by HS in Cappelli, while in Ofanto the level of transcription was much lower. The same pathway is activated in both cultivars under CS. A representation of the β-oxidation pathway with indicated variations in the expression levels of the genes coding for the key enzymes of the pathway is reported in Figure 4.

### The glyoxylate cycle

As β-oxidation pathway, also glyoxylate cycle occurs in peroxisomes and in glyoxysomes. In the glyoxysomes, acetyl-CoA is not further oxidized to produce energy and it is used as substrate to produce sugars. The reactions that occur in this pathway are known as glyoxylate cycle. In the glyoxysome, through a β-oxidation process, the fatty acids are converted into acetyl-CoA units and the reactions are catalyzed by well-known enzymes [48]. The expression level of the gene *At3g16910* coding the enzyme named acyl-activating enzyme 7 (AAE7; EC 6.2.1.1), responsible for the acetyl-CoA synthesis, is described by four probe sets and, among these, one belongs to cluster 1 (TaAffx.31783.1.S1_at, Additional file 5) while the other three were not differentially expressed. The first step of the glyoxylate cycle is catalyzed by the citrate synthase enzyme (CS, EC 4.1.3.7). On the array there are three probe sets which show a sequence similarity with *Arabidopsis* citrate synthase gene (*At2g42790)* and, among these, one belongs to cluster 17 (Ta.970.2.S1_a_at) while the other two were not differentially expressed. Subsequently the citrate is converted to isocitrate by a two-step reaction catalyzed by aconitase (ACO, EC 4.2.1.3). Among the twenty probe sets with high sequence similarity to aconitase genes, only one is differentially expressed (TaAffx.6397.1.S1_at) and belongs to cluster 30. The expression of the isocitrate lyase (ICL, EC 4.1.3.1) gene (*At3g21720*) responsible for the third step of the cycle is described by the TaAffx.79139.1.S1_at probe set that belongs to cluster 17. There are three other different probe sets that show high sequence similarity to *At3g21720*, but they were not differentially expressed. Five probe sets represent the gene (*At5g03860*) for the malate syntase (MS, EC 2.3.3.9) that catalyze the fourth step of the cycle. Among these one (Ta.23970.1.A1_at) was differentially expressed and has been classified in cluster 17, another (Ta.23970.1.A1_x_at) has not been assigned to any cluster, and the remaining three were not differentially expressed (Additional file 3). The final step of glyoxylate cycle is catalyzed by malate dehydrogenase. In *Arabidospsis* there are 10 genes coding malate dehydrogenases (MDH, EC 1.1.1.37) that act in cytoplasm, mithocondria, chloroplasts and glyoxysomes. The wheat GeneChip^®^ carries 48 probe sets and only one of them is differentially expressed (Ta.25543.2.S1_at) and belongs to cluster 17.

**Figure 5:**
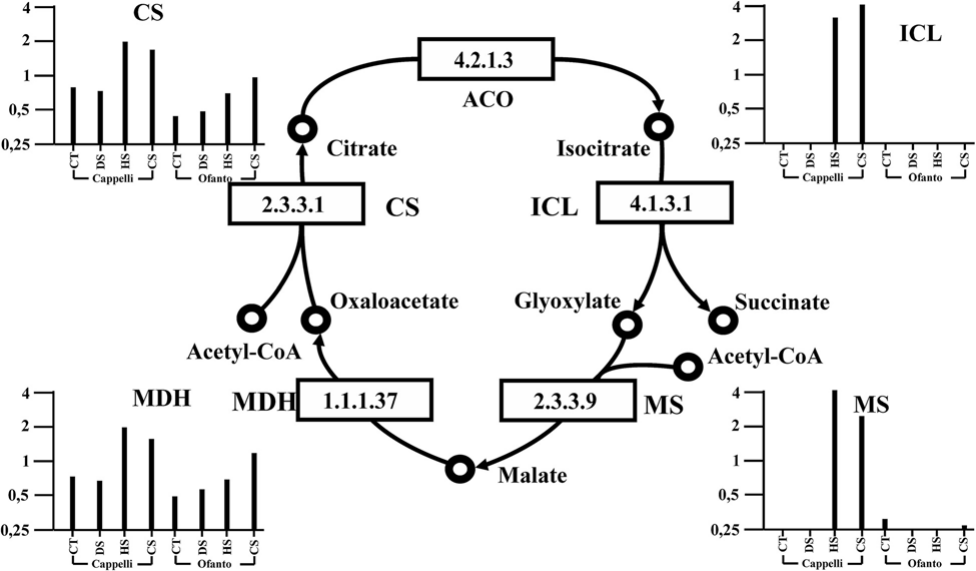
**Representation of the glyoxylate pathway: variations in the expression levels of the genes coding for the key enzymes.** The four treatment conditions, grouped by genotypes, are plotted on x axis. The relative expression level (data normalized to the median for each probe set) is plotted on the y axis. ACO: aconitase (EC 4.2.1.3). CS: citrate synthase (EC 2.3.3.1); ICL: isocitrate lyase (EC 4.1.3.1); MDH: malate dehydrogenase (EC 1.1.1.37); MS: malate syntase (EC 2.3.3.9). CT = Control samples; DS = Drought stressed samples; HS = Heat stressed samples; CS = Combined stress samples.

Similarly to the fatty acid β-oxidation pathway, the glyoxylate cycle appear to be activated by HS specifically in Cappelli, and by CS in both genotypes. A representation of the glyoxylate cycle illustrating the variations in the expression levels of the genes coding for the key enzymes of the pathway is reported in Figure 5.

### Senescence marker genes

The activation of the glyoxylate cycle genes is considered a clear sign of plant cell senescence [49, 50]. To study putative correlation among glyoxylate cycle activation and senescence in durum wheat, several genes, known as senescence markers [51], were checked. A NAC transcription factor (*At5g39610*, *NAC6*/*ANAC092*) was already described as a gene involved in salt promoted senescence [52] and in this experiment it has a cluster 26 expression trend. In *Arabidopsis* the gene *At3g56400* (*WRKY70*) is a negative regulator of senescence [53]. The wheat GeneChip^®^ has three probe sets with high sequence similarity to *WRKY70* and one of them was down-regulated with an opposite expression trend if compared to glyoxylate cycle genes. A third transcription factor involved in senescence processes (*TaNAM-B1*) was found differentially expressed and belongs to cluster 4. *TaNAM-B1* and its homologs are responsible for senescence processes in wheat [54].

Although our data do not show a wide activation of senescence-related genes, several senescence transcription factors were clearly activated with an expression trend correlated to glyoxylate cycle and to many stress response genes, indicating the activation of early heat stress-induced senescence in Cappelli.

### Modulation of gene expression induced by drought at tillering stage and e-QTL mapping

**Figure 6:**
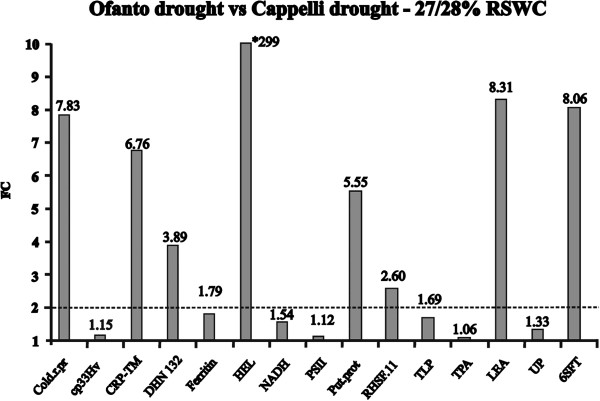
**Fold Change values (FC, number on each column) of the expressed genes selected based on the array hybridization data of flag leaves at booting stage and tested in leaves of drought treated young plants at tillering stage by qRT-PCR analysis.** The FC value is the ratio of the expression level of the selected genes in the comparison Ofanto *vs* Cappelli samples subjected to severe drought stress of 27/28% Relative Soil Water Content (RSWC). The acronyms of the genes in the figure mean: Cold.r.pr (Cold regulated protein, Ta.13183.1.S1_x_at); Cp33Hv (RNA-binding protein cp33 precursor–barley, Ta.18434.1.S1_at); CRP-TM (cold regulated protein, Ta.13183.1.S1_s_at); DHN 132 (dehydrin, DHN, Ta.13255.1.S1_at); Ferritin (Ferritin, Ta.681.2.S1_a_at); HEL (High Expression Level; TaAffx.100436.1.S1_at); NADH (NADH dehydrogenase subunit B, TaAffx.112816.1.S1_at); PSII (Photosystem II 10 K protein precursor; Ta.28750.2.A1_x_at ); Put. Prot (Putative protein, Ta.29464.1.A1_at); RHSF.11 (heat shock factor RHSF11, TaAffx.34778.1.S1_at); TLP (thaumatin-like protein, Ta.25053.1.S1_at); TPA (transposase, Ta.3145.1.S1_at); LEA (Late Embryogenesis Abundant protein, Ta.5913.1.S1_at); UP (Unknown Protein, Ta.28273.1.S1_x_at); 6-SFT (Sucrose:fructan 6-fructosyltransferase, Ta.2789.2.S1_at). * this peak was corrected for comparison to the others.

The comparisons of the transcriptomic data as well as the QT-clustering experiment have indicated a different strategy in the response of Cappelli and Ofanto to drought and heat stress at booting stage. Since the difference in WUE between the two cultivars is manifested constitutively through all life cycle [17], 15 drought responsive genes at booting stage (10 more expressed in Ofanto DS *vs* Cappelli DS and 5 *vice-versa*) were also tested in drought treated plants at tillering stage. All genes confirmed their drought responsiveness in one or in both cultivars. In each cultivar, 12 genes induced by drought were found. The comparison of the expression level of the genes in Ofanto drought *vs* Cappelli drought highlighted 8 genes with a significantly higher expression level in Ofanto *vs* Cappelli (fold change > 2), while the remaining 7 genes, although generally more expressed in Ofanto, were not significantly different (Figure 6, Additional file 6). These findings suggest that at tillering stage the response of Ofanto to drought is stronger, in terms of gene expression levels, than the response of Cappelli to the same condition. This results, once again, highlights the different response strategy of these two cultivars to drought conditions with a stronger molecular response in Ofanto that is, to some extent, coherent with the results of the transcriptomic analysis performed at booting stage.

To gain more details on these contrasting responses we have taken advantage of the genetic tools available for this couple of genotypes and we tested the possibility of mapping QTLs controlling the expression level (e-QTLs) of genes differentially expressed between the cultivars tested. The experiment was carried out using the gene corresponding to the probe set TaAffx.100436.1.S1_at (annotated as High Expression Level gene, *HEL*). At booting stage, the microarray data indicate that this gene was expressed in Ofanto and up-regulated in response to all stress treatments, while an expression below the background value was detected in Cappelli in all tested conditions (Additional file 3). When the expression profile of *HEL* has been verified in an independent experiment carried out at tillering stage in plants subjected to severe drought stress (27-28% RSWC), the qRT-PCR analysis have revealed that *HEL* is clearly expressed in both cultivar in the absence of stress (Ctrl) and down-regulated in response to drought treatment. Nevertheless, the *HEL* transcription level resulted to be much lower in Cappelli than in Ofanto (Additional file 6), so that the fold change value of the ratio Ofanto drought *vs* Cappelli drought was 299-fold higher in Ofanto with respect to Cappelli (Figure 6). This great difference in gene expression associated to drought response was considered an ideal requirement to identify e-QTLs controlling drought gene expression.

The sequence of *HEL* transcript was obtained after “blastn” submission of the TaAffx.100436.1.S1_at probe set in CerealsDB database (Chinese spring Contig444272, 1,448 bp), TIGR Plant Transcript Assemblies (TA97230_4565) and DFCI Wheat Gene Index database (TC419595). A research for ortholog genome sequences in close related *Brachypodium* and *Oryza* species, a search for coding region detection in DNA sequences using ESTScan as well as an analysis for protein sequence in UniProt did not provide any information about these sequences annotation and function. Otherwise, alignment of these sequences detected a putative intron (Additional file 7).

**Figure 7:**
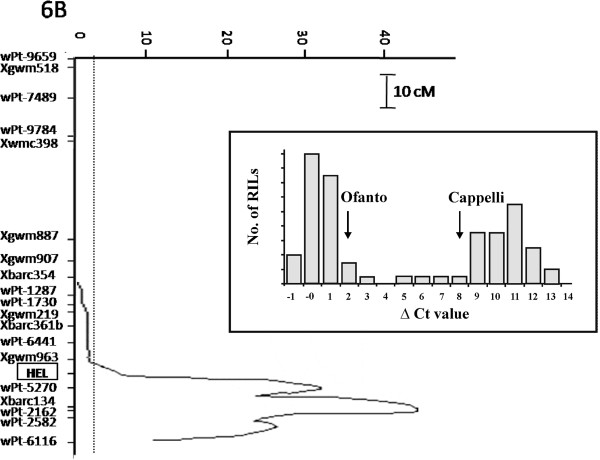
**Partial genetic map of the region of the durum wheat chromosome 6B (long arm) carrying the** ***HEL locus,*** **the major QTL controlling** ***HEL*** **expression level and the corresponding curve of LOD values.** The insert reports the frequency distribution of ∆Ct values of *HEL* expression detected in 80 RILs deriving from the cross between Ofanto and Cappelli and subjected to drought stress (tillering stage) determined by a relative soil water content (RSWC) of 27-28%.

The accumulation of *HEL* transcripts in response to drought was tested on 80 Recombinant Inbred Lines (RILs) from the cross Ofanto x Cappelli exposed to drought at tillering stage. The expression data showed a bimodal frequency distribution in the RIL population that indicates a simple genetic control of the trait (Figure 7). When the Ofanto x Cappelli genetic map, also including the marker corresponding to the *HEL* gene, was used to perform the e-QTL analysis, a single e-QTL with a strong effect located in the telomeric region of the long arm of chromosome 6B was detected. The e-QTL showed a LOD of 43.9, with 95.2% of the observed variability explained, and a negative additive effect was found (-4.93), which indicated that the allele carried by Ofanto was effective in increasing the *HEL* expression level. Notably, the e-QTL was positioned in the same chromosomal region where the *HEL* gene was mapped (Figure 7), a finding suggesting that the main factor controlling *HEL* expression might rely in the gene sequence itself.

## Conclusions

### Ofanto and Cappelli: two different stress responsive strategies

The durum wheat cultivars Ofanto and Cappelli represent an ideal experimental system to investigate the water stress response in durum wheat. The two cultivars are characterized by a significantly different WUE [16, 17], an Ofanto x Cappelli RIL population has been implemented with a molecular marker map [19, 20] and it is currently used to map some WUE-related traits [18]. The present work was designed to provide an additional level of knowledge on the Ofanto and Cappelli experimental system through a transcriptomic analysis of the molecular response to drought, heat, and a combination of both stress conditions.

The transcriptomic data highlight that Ofanto activated a large set of well-known drought-related genes after drought treatment, while Cappelli showed the constitutive expression of several genes that in Ofanto are induced by drought and a minimal modulation of gene expression in response to stress. Assuming that the extent of gene modulation (number of genes modulated in response to stress) is a consequence of the stress signal perception, the same experimental conditions, determined as SWC, had a different impact both on RWC and on stress signaling in Cappelli and Ofanto. Despite the lower RWC of Cappelli compared to Ofanto, the former cultivar showed a minimal gene activation in response to drought. The lower stomata conductance [17] and the constitutive expression of some drought-related genes might contribute to limit the effect of drought and the stress perception in Cappelli which, in turn, is reflected in a minimal drought-induced gene expression.

The heat response was characterized by the remarkable induction, in Cappelli only, of the genes coding for the enzymes involved in the fatty acids β-oxidation and in the glyoxylate cycle. An enzyme of the β-oxidation pathway, the thiolase (AAT, EC 2.3.1.16), is also involved in ABA signaling. Jiang *et al*. [55] have provided genetic evidence that thiolase positively regulates ABA signaling, including ABA-induced stomata closure in *Arabidopsis*. Overexpression of the gene coding thiolase increased biosynthesis of jasmonic acid (JA) and accelerated dark-induced senescence of leaves; the opposite characteristics were observed in antisense transgenic lines [56]. The enhanced expression of the thiolase gene, together with the activation of the glyoxylate cycle genes (generally associated with senescence [49, 50]) and with the modulation of some senescence marker genes, might suggest an early activation of the senescence and of senescence-related processes in Cappelli but not in Ofanto.

The combination of drought and heat stress in plants is a unique stress sharing a marginal portion of the molecular responses activated by drought and heat stress alone [12–15, 57–59]. This is evident in the case of the cultivar Ofanto that activates 707 PSs in response to drought stress, 3,243 in response to heat stress and 5,645 in response to drought and heat combined stress. However we observed the opposite phenomenon in Cappelli, since it modulates 248 PSs in response to drought stress, 3,852 PSs in response to heat stress and “only” 1,814 in response to the drought and heat combined stress.

The great differences found in the stress response between Ofanto and Cappelli at transcriptomic level underline that cultivars of the same species can use different molecular strategies to cope with unfavorable climatic conditions. Other examples are also known; the different level of salt-tolerance between FL478 (tolerant) and IR29 (sensitive) rice varieties was associated to a different expression of transcripts corresponding to cation transport proteins, involved in reducing Na^+^ influx, and enzymes with antioxidant activity as peroxidases [60, 61]; the different heat tolerance of TAM107 (tolerant) and Chinese Spring (sensitive) bread wheat cultivars was associated to 313 genes differentially expressed between the two varieties in response to heat stress [10].

### e-QTL analysis in young plants

Many studies are available in literature about linkage mapping of genomic regions involved in the control of agronomic traits, but few are still the evidences about e-QTLs [62–64]. A pioneering study in this field was represented by the e-QTL analysis of *Cor14*, a cold regulated gene of wheat and barley. *Cor14* gene expression was higher in frost resistant *vs* susceptible cultivars and the QTL analysis identified a single e-QTL co-segregating with a frost tolerance QTL and with the *CBF locus*, coding key CBF transcription factors controlling the cold response [65, 66]. In this study a similar approach was carried out with a drought induced gene (*HEL*) showing a marked difference in expression level between Ofanto and Cappelli exposed to drought. The expression of a gene can be controlled by a number of different factors, from epigenetic to translational level. In general, *cis*-acting factors, like features of promoters or introns as examples, or trans-acting factors, as transcriptional regulators or protein acting on mRNA splicing, translation or stability of transcripts, can influence the accumulation of the mRNA of a gene [67]. The fact that the analysis of *HEL* gene expression has revealed a single e-QTL with a strong effect (more than 90% of explained phenotypic variability) coincident with the *HEL locus* strongly suggests that the main factor controlling its expression relies in the gene sequence itself. Otherwise, if a trans-acting factor is somehow involved, we can conclude that it is located in the same genomic *locus* of the *HEL* gene. However, the transcription profiles of 41 double haploid wheat lines using the Affymetrix GeneChip^®^ Wheat Genome Array have revealed the putative position of 542 genes identified as major e-QTLs on single-chromosomal regions [68].

Some QTLs for traits related to grain yield in dry environments were previously identified in the region of the e-QTL found in this study, based on molecular markers in common with published genetic maps [69, 70]. Since large parts of the wheat genome are covered with QTLs for drought-related traits, a genetic association between a QTL and an e-QTL is not sufficient to infer for a role of the gene in drought tolerance, but it can represent an indication to be tested with further experiments.

The mapping of the e-QTL controlling *HEL* gene expression, together with other experiments here reported, provides clear evidences that the genetic system based on Cappelli and Ofanto represents an ideal tool for the genetic dissection of the molecular response to drought and other abiotic stresses.

## Methods

### Plant material and growth conditions

For transcriptomic analysis at booting stage two *Triticum turgidum* subsp*. durum* cultivars, Ofanto and Cappelli, were grown in a growth chamber under conditions mimicking the natural growing season. Germination was carried out at 6°C in complete darkness until shoot emergence, then the growth conditions were set at 10 hrs light and 14 hrs dark at 8°C and 6°C, respectively, relative humidity 60%. Growing conditions were then modified according to the plants growth stage: 13 hrs light and 11 hrs dark with 12°C and 8°C, respectively, relative humidity 50% at second leaf stage; 14 hrs light and 10 hrs dark with 15°C and 10°C, respectively, relative humidity 50%, at the third leaf stage; 15 hrs light and 9 hrs dark with 15°C and 12°C, respectively, relative humidity 50%, at tillering stage; 15 hrs light and 9 hrs dark with 19°C and 15°C, respectively, relative humidity 40% at booting stage. Light was provided by 400-W high-pressure lamps (Philips) with a photon flux density of 500 μmol m^-2^ s^-1^.

Plants were grown in pots (38 × 16 cm, 14 cm depth, 12 seeds/pot) filled with a substrate of soil (clay-loam), sand and peat in a 6:3:1 ratio, respectively. Pots filled with the substrate were standardized for weight and field capacity. A complete randomized block design with three replications was adopted. Ammonium biphosphate (pre-sowing) and ammonium nitrate (shoot emergence and tillering) were used as fertilizers for a total amount equivalent to 60 kg/ha of P and 150 kg/ha of N. Powdery mildew was controlled with fungicide (Folicur 1.5 g/L) from tillering onwards. The irrigation was made with tap water (0.5 dS m-1), weighting pots and intervening whenever the soil lost 50% of available water, bringing it back to a soil water content (SWC) corresponding to 28%.

Drought stress (DS), heat stress (HS) and combined stress (CS) were imposed simultaneously when the plants reached the booting stage. Control (Ctrl) plants were kept in the conditions described above and watered to maintain SWC equal to 28%. DS was imposed by withholding water and allowing the pots to reach SWC equal to 12.5%. To monitor SWC the pots weight was measured twice per day. The flag leaves detached at mid of the light period were collected for transcriptomic analysis three days after the imposition of stress. HS was imposed by increasing the temperature for two days at 30°C and 22°C in the 15 hrs light and 9 hrs dark regime, for an initial adaptation phase, then the temperature was increased to 34°C and 26°C light/dark for two days and subsequently to 40°C and 32°C light/dark for an additional day when the flag leaves were collected. CS was imposed withholding water to reach SWC equal to 12.5% as described for DS, and increasing the temperature up to 40°C as described for HS. Before leaf sampling, the flag leaf temperature was evaluated using the Testo-925 thermometer (Table 1). The plant water status was monitored as Relative Water Content (RWC%) determined on the same plants used for RNA isolation using the leaf below the flag leaf (Table 1). All experiments were carried out in triplicate. Leaf material used for molecular analysis was immediately frozen in liquid nitrogen and kept at -80°C.

An additional transcriptomic analysis was carried out at tillering stage. Seedlings of Ofanto, Cappelli and 80 Recombinant Inbreed Lines (RILs) from the cross Ofanto x Cappelli were transferred to pots of 9.5, 7.0 and 17 cm, top diameter, bottom diameter, and depth, respectively (one plant for each pot) at Zadoks stage 0.7 [71], i.e. when the coleoptile had emerged from the caryopsis. The plants were grown in a growth chamber, at 13 hrs light and 11 hrs dark at 20°C and 15°C respectively, with average quantum flux density of PAR of 400 μmol m^-2^ sec^-1^ and humidity of 70% (day) and 90% (night). Before planting, the soil dry weight (DW) and the weight of soil watered till field capacity (WFC) were measured. Relative soil water content (RSWC) was calculated as: RSWC = ((current pot weight – DW)/(WFC-DW))*100. A slow water stress was applied by withholding water for 23 days starting at stage DC14-19 (Zadoks scale) and approximately till tillering (Zadoks stage DC22-29) when the final RSWC level was 27-28% which corresponds to a severe stress. Pots were weighted daily and control plants were watered to 95% of RSWC continuously. The uppermost fully expanded leaves were harvested all at the same time and used for RNA isolation.

### RNA isolation and array hybridization

Total RNA was extracted from leaf tissues by TRIZOL reagent according to the method published by the *Arabidopsis* Functional Genomics Consortium http://www.arabidopsis.org/portals/masc/AFGC/RevisedAFGC/site2RnaL.htm#isolation. RNA was cleaned using RNeasy columns according to the Qiagen RNeasy Mini Handbook. To assess RNA quality and quantity, several dilutions of each sample were analysed using the Agilent RNA 6000 nano Kit and Agilent Bioanalyzer 2100. RNA samples were processed following the Affymetrix GeneChip^®^ Expression Analysis Technical Manual (Affymetrix). Single-stranded, then double-stranded cDNAs were synthesized from the poly(A) mRNA isolated from 5 μg of total RNA for each sample using the Affymetrix One-Cycle Labeling kit and Control reagents. The resulting *ds-*cDNA was column purified and then used as a template to generate biotin tagged cRNA from an *in vitro* transcription reaction (IVT), using the Affymetrix GeneChip^®^ IVT Labelling Kit. Fifteen μg of the resulting biotin-tagged cRNA was fragmented to strands of 35–200 bases in length following prescribed protocols (Affymetrix GeneChip^®^ Expression Analysis Technical Manual) and then hybridized at 45°C with rotation for 16 h (Affymetrix GeneChip^®^ Hybridization Oven 640) to probe sets present on an Affymetrix GeneChip^®^ Wheat Genome Array. The arrays were washed and then stained (SAPE, Streptavidin-phycoerythrin) on an Affymetrix Fluidics Station 450 followed by scanning with a GeneChip^®^ Scanner 3000. Wheat microarray design and expression profiling data are available in PlexDB (http://www.plexdb.org) [72] as experiment “TA47” and at http://www.ncbi.nlm.nih.gov/geo as experiment GSE45563.

### Data processing and analysis

GeneChip^®^ hybridization quality was ensured using the standard Affymetrix controls. B2 oligonucleotides were spiked into each hybridization cocktail. PolyA controls (*lys, phe, thr, dap*) and hybridization controls (*BioB, BioC, BioD* and *Cre*) were used to monitor the labelling and hybridization processes. To draw the “RNA degradation plot” relative to probe signal intensities of the *actin* and *GAPDH* control genes, the R “simpleaffy” library was used. Raw intensity values were normalized by RMA (Robust Multi-array Average) [73] using the R package “Affy” [74]. The same package was used to run the MAS 5.0 algorithm on raw data to produce a detection call for each probe set. These detection calls ("present", "marginal" or "absent") were used to apply an initial filtering step, since genes not expressed ("absent") represent experimental noise and can generate false positives. We removed from analysis all the probe sets that did not show all the three "present" calls in at least one sample. R-squared linear correlation coefficients were computed on the RMA expression values (log2-transformed) for each set of biological triplicates. RMA filtered data were imported to the software Genespring GX 7.3 (Agilent Technologies) and all subsequent analyses were carried out with this software.

Differentially expressed probe sets were identified through a Welch t-test with Benjamini and Hochberg false discovery rate correction for multiple tests [23]. Differences in gene expression were considered to be significant when p-value was lower than 0.01 and induction or repression ratio was equal or higher than 2-fold. Principal Component Analysis (PCA, [75]) was employed to assess the contribution of genotype and treatment factors in the variation detected in the dataset. Clusters of genes with distinctive expression patterns were searched with QT (Quality Threshold) cluster analysis [76]. QT clustering algorithm groups genes into high quality clusters based on two parameters: "minimum cluster size" and "minimum correlation". The minimum cluster size was set to 30 and minimum correlation to 0.80. Functional gene categories over-represented in the clusters in comparison with the whole microarray were searched at the MIPS *Arabidopsis thaliana* database (MAtDB) Functional Catalogue (FunCat, http://mips.helmotz-muenchen.de/proj/funcatDB). MIPS FunCat is a hierarchical database that links *Arabidopsis locus* identifiers to functional categories. The FunCat database currently contains 28 main categories subdivided into 1,289 subcategories [77]. Blast searches were done using HarvEST: Affymetrix Wheat1 Chip 1.50 (http://www.harvest.ucr.edu) and only the annotations of wheat probe sets with a homology level cut-off equal or lower than E-value = e^-10^ were considered.

### qRT-PCR analysis of drought-responsive genes and expression-QTL mapping at tillering stage

Total RNA was isolated from well-watered leaves and drought stressed leaves using the TRI Reagent^®^ Solution (Ambion) and the Qiagen RNA Cleanup Protocol (RNeasy plant mini kit Qiagen). DNA contaminations were removed with the Ambion’s DNA-*free*™ Kit. RNA quantity and integrity was confirmed loading the purified RNAs onto an Agilent^®^ RNA Nano 6000 chip and analyzed on an Agilent^®^ 2100 BioAnalyzer according to the manufacturer’s instructions. The cDNA was synthesized with Standard Reverse Transcription Protocol (Promega) and quantified by Qubit fluorometer™ (Invitrogen).

The description of the selected genes and the complete set of primer pairs are listed in Additional file 8. The specificity and uniqueness of the primers and the amplicons were verified by amplicon sequencing. qRT-PCR reactions were performed with SYBR Green fluorescence detection in a qPCR thermal cycler (ABI PRISM 7300, Applied Biosystems). Each reaction was prepared using 3 μL from a 2 ng/μL dilution of cDNA derived from the RT reaction, 12.5 μL of SYBR Green PCR Master Mix (Qiagen), 1 μM forward and reverse primers, in a total volume of 25 μL. The cycling conditions were: 10 min at 95°C, followed by 40 cycles of 95°C for 15 sec and 60°C for 1 min with the final dissociation at 95°C for 15 sec, 60°C for 30 sec and 95°C for 15 sec. In order to identify a reference gene, the algorithm described by Aprile *et al*. [7] was used to find stably expressed transcripts using microarray data set. The best three ranking probe sets based on Coefficient of Variation (CV) and expression level were: Ta.12727.1.S1_at (CV = 0.075; *Arabidopsis* homolog = *At2g42210*, *OEP16-3*), Ta.9617.1.S1_at (CV = 0.090, *At1g16700*, *NADH-ubiquinone oxidoreductase*) and Ta.3583.1.A1_at (CV = 0.066, *At1g69120*, *AGL7*). The CVs based on qRT-PCR data were: CV_*OEP16-3*_ = 0.018, CV_*NADH-ubiquinone* oxidoreductase_ = 0.020, CV_*AGL7*_ = 0.033. The CVs were lower than 0.050 and suitable as qRT-PCR reference genes. In the present work, we used *OEP16-3* as reference gene since it had the lowest CV. Three biological replicates were used for quantification analysis. Melting curve analysis was performed to evaluate the presence of non-specific PCR products and primer dimers. Comparative C_t_ method calculation steps to calculate the fold changes (FC) consist of the following three steps: 1) normalization to endogenous control by comparison of target gene and endogenous control ∆C_t_ = C_t target gene_- C_t endogenous gene_; 2) normalization to calibrator sample ∆C_t sample_-∆Ct_calibrator_ = ∆∆C_t_; 3) using the formula 2^-∆∆Ct^.

To validate the microarray data, four probe sets were subjected to real-time qRT-PCR analysis: Ta.9335.1.S1_at (Acyl-CoA Oxidase, *ACX*), Ta.9184.1.S1_at (Multienzymatic complex, *ECH/HCD*), Ta.28367.1.S1_at (Acetyl-CoA acyltransferase, *AAT*), TaAffx.31738.1.S1_at (Acetate-CoA ligase/synthase, *ACL*).

The transcript *HEL* (High Expression Level) corresponding to the TaAffx.100436.1.S1_at probe set was selected as the most differentially expressed between Ofanto and Cappelli. The probe set sequence was used as a query in a blastn search against the Chinese Spring genomic DNA database of Cerealsdb database (http://www.cerealsdb.uk.net/CerealsDB/Documents/DOC_search_reads.php) and the clone Contig444272 (1448 bp) was obtained (the sequence information are in Additional file 9). Two primers designed on Contig444272 (5’-GTGGGTGGAGTGCATGTGGGTT-3’ and 5’-ACCGGCGTTATCTGCGGTTGC-3’) amplified a fragment of near 1000 bp in Ofanto only, while no amplicons were found from genomic DNA of Cappelli. Amplicon identity was confirmed by sequencing. The PCR reactions were performed in 25 μL volume with 80 ng of template DNA, 1.5 mM MgCl_2,_ 0.2 mM of dNTPs, 1x PCR-Buffer, 0.2 μM of the two primers, and 1 U of Platinum Taq DNA polymerase (Invitrogen); annealing temperature was set at 63°C. This polymorphism was employed to map the corresponding *locus* in the frame of the previously published Ofanto x Cappelli genetic map [19, 20], using the Kosambi mapping function within the JoinMap 4 software [78], considering a minimum LOD score (log_10_ of the odds ratio) of 4.0.

The expression of the gene corresponding to TaAffx.100436.1.S1_at probe set was tested in response to drought on 80 Recombinant Inbreed Lines (RILs), representing the genetic variability of Ofanto x Cappelli segregating population. Two replications were considered for each RIL. QTL analysis was performed using the software package MapQTL^®^, version 5.0 [79] and the genetic map described by Marone *et al*. [19, 20]. The logarithm of odds (LOD) profiles from simple interval mapping were inspected, and the marker closest to each LOD peak was selected as the cofactor to perform the multiple QTL mapping analysis. The LOD significance threshold levels of the respective traits were calculated with the permutation test option provided in MapQTL, using 10,000 permutations.

## Electronic supplementary material

Additional file 1: **RNA degradation plot of the reference probe sets relative to GAPDH gene.** On x-axis there are the 11 probes sorted by position along the *GAPDH* gene (from 5’ to 3’). On y-axis is reported the relative expression value (scaled and shifted). Parallel lines mean that the RNA degradation is constant among samples. (PPTX 189 KB)

Additional file 2: **Validation of microarray data for four probe sets by qRT-PCR.** In each graph the array data are plotted on the left and qRT-PCR data on the right. Blue bars represent Ofanto data. Red bars represent Cappelli data. CTRL expression data were used as baseline for fold change calculations. The log2 fold change is plotted on the y-axis. The Pearson correlation among microarray FC and qPCR FC is 0.753. ACS: acyl-CoA synthase; ACX: acyl-CoA oxidase; ECH: enoyl-CoA hydratase; HCD: L-β-hydroxyacyl-CoA dehydrogenase; AAT: acyl-CoA-acetyltransferase enzyme or thiolase. (PPTX 130 KB)

Additional file 3: **The list of the 9,012 differentially expressed genes in the experiment is sorted in column A.**  For each probe set the relative homologous *Arabidopsis* gene (column B), e-score (C), annotation (D), the mean expression level for each treatment (columns E-L) are reported. From column M to column V are reported the statistical comparison results. Empty cells mean not differentially expressed. Column W shows the QT-cluster number for each probe set. (XLSX 2 MB)

Additional file 4: **QT-clustering analysis obtained using the expression values of the 8,660 stress-related genes differentially expressed in at least one condition/genotype.** The analyses was performed with a minimum cluster size of 30 and a correlation value of 0.80. The four treatment conditions, grouped by genotypes, are plotted on x axis. The relative expression level (for each probe set the data were normalized to the median expression level of the Ofanto Ctrl samples) is plotted on the y axis. The lines represent the mean expression trend of all probe sets belonging to each cluster. (PPTX 479 KB)

Additional file 5: **The list of the 1,923 differentially expressed genes belonging to cluster 1, 4, 8, 10, 17, 18, 23, 40 and 47 sorted by QT-cluster number (column W).** For each probe set the relative homologous *Arabidopsis* gene (column B), e-score (C), annotation (D), the mean expression level for each treatment (columns E-L) are reported. From column M to column V are reported the statistical comparison results. (XLSX 426 KB)

Additional file 6: **Data from Affymetrix hybridization experiments expressed as Fold Change (FC) in black. In comparison with the FCs of microarray data, the FCs of qRT-PCR results from the experiment of young plants at tillering stage subjected to severe drought stress of 27/28% of RSWC are reported in blue.** The results showed the differences in gene expression between Ofanto and Cappelli in non stressed/control (Ctrl) *vs* stressed/drought (Dr) condition in 3rd and 4th columns, while the FC values in the comparisons Ofanto stressed/drought (Dr) vs Cappelli stressed/drought (Dr) and Ofanto non stressed/control (Ctrl) vs Cappelli non stressed/control (Ctrl) are listed in the 5th and 6th columns. (DOCX 24 KB)

Additional file 7: **Alignment analysis of Contig444272 sequence (1,448 bp) with the homologous sequences i) Plant Transcript Assembly TA97230_4565 Sequence, Accession TA97230_4565** ***Triticum aestivum*** **ii) TC419595 (from DFCI Wheat Gene Index, Release12.0) iii) probe set TaAffx.100436.1.S1_at of Wheat Affymetrix Chip.** From the alignment, carried out using the AlignXprogramm (VectorNTI, Invitrogen), the results suggest that Contig444272 sequence contains an intron in the region from 372 to 493 bp. (DOCX 175 KB)

Additional file 8: **Primer lists used to find expression level polymorphisms and to validate microarray data.** (DOCX 19 KB)

Additional file 9: **Nucleotide sequence (223 bp) of** ***HEL*** **gene obtained from cDNA of Ofanto and Cappelli by sequencing analysis in the first part of the table, and in the second part Contig444272 sequence (1448 bp) of Chinese spring obtained from cerealsDB database.** (DOCX 17 KB)
