# Supplementary figures and images for: Different stress responsive strategies to drought and heat in two durum wheat cultivars with contrasting water use efficiency
Source: BMC Genomics. 2013 Nov 22;14(1):821. doi: 10.1186/1471-2164-14-821 (PMC4046701; doi:10.1186/1471-2164-14-821)

## Slide 1
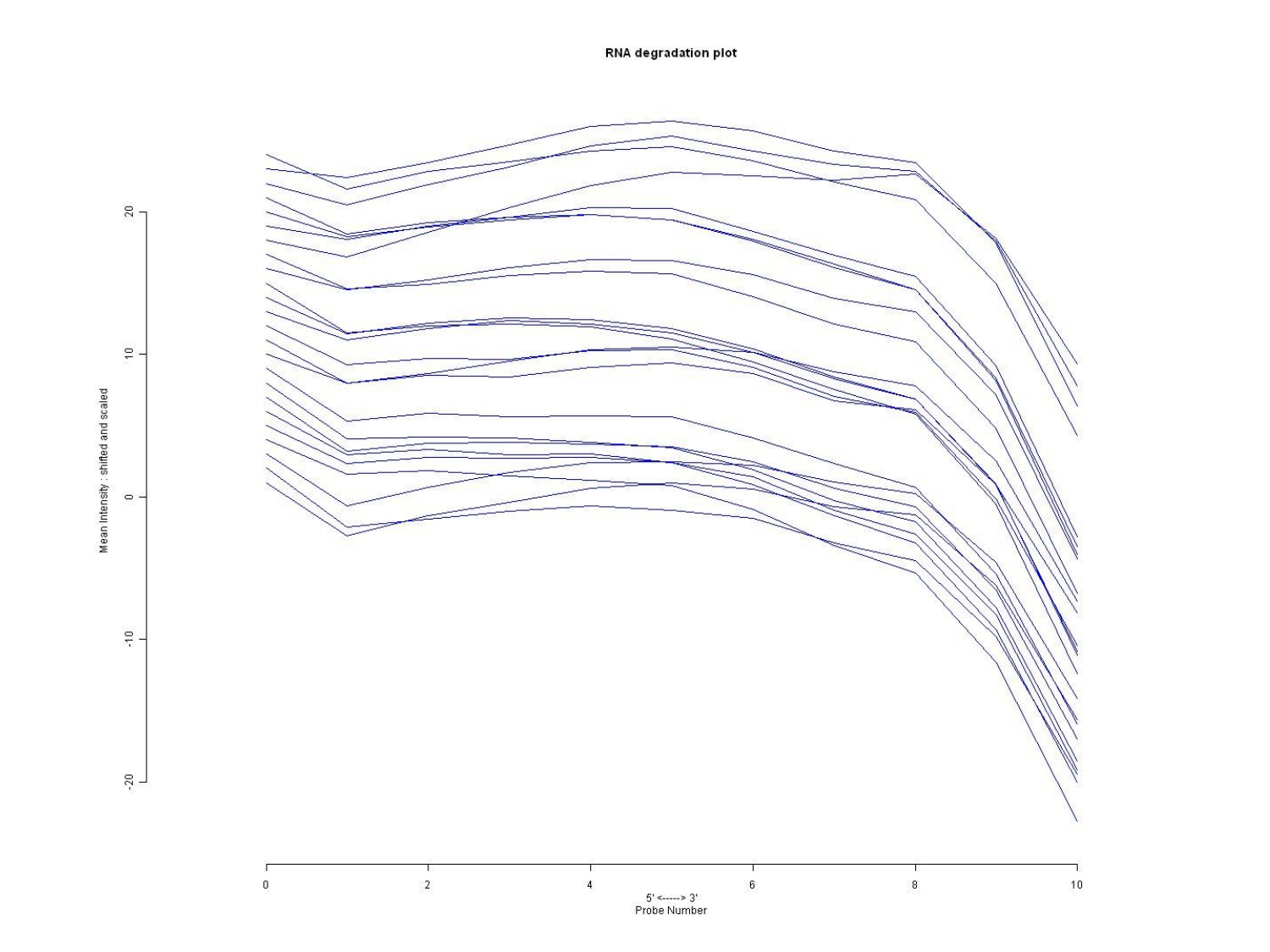

Supplement: Supplementary file 1 — Additional file 1: RNA degradation plot of the reference probe sets relative to GAPDH gene. On x-axis there are the 11 probes sorted by position along the GAPDH gene (from 5’ to 3’). On y-axis is reported the relative expression value (scaled and shifted). Parallel lines mean that the RNA degradation is constant among samples. (PPTX 189 KB) [file 12864_2013_5521_MOESM1_ESM.pptx]

## Slide 1
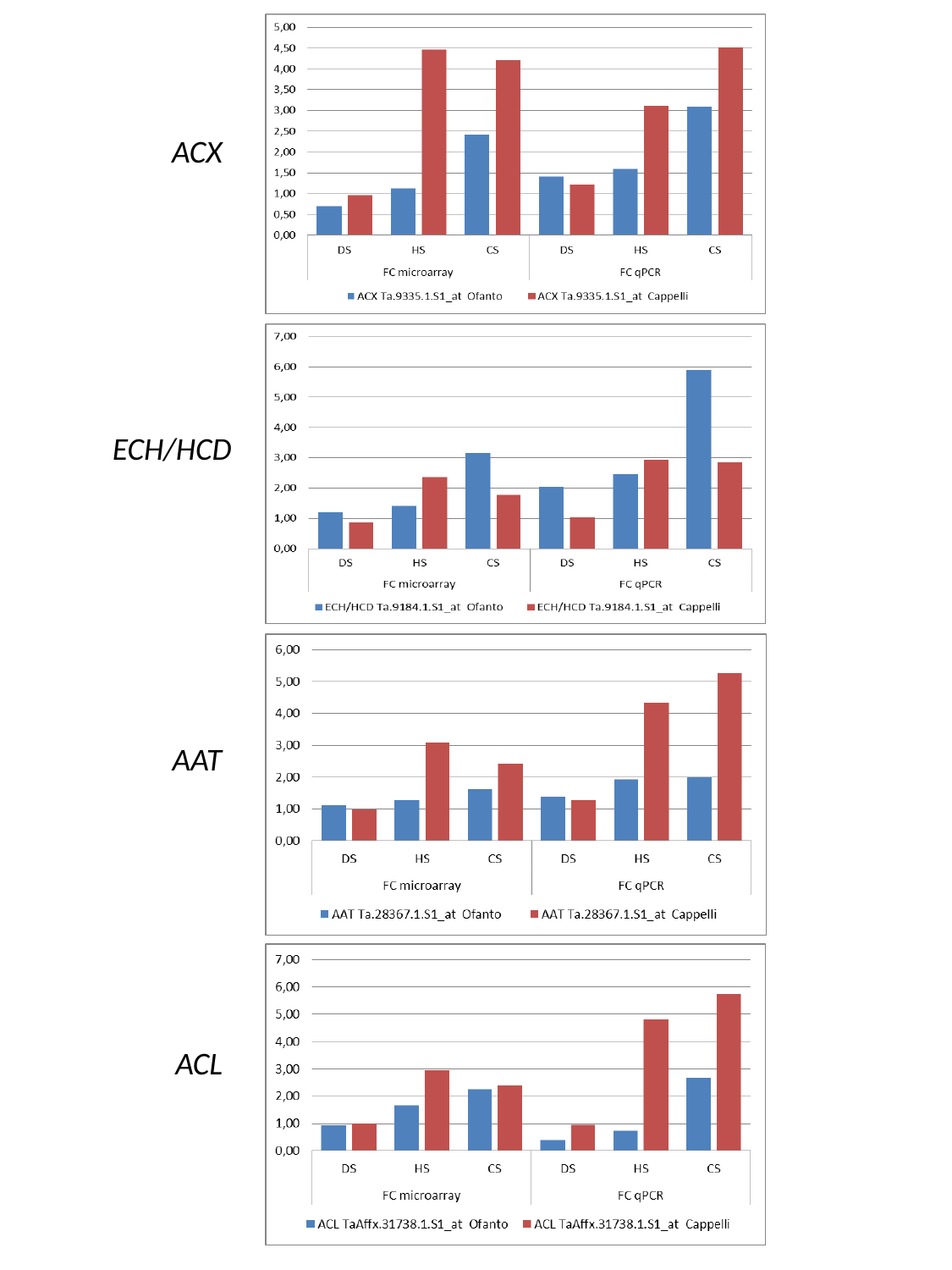

ACX
ECH/HCD
AAT
ACL

Supplement: Supplementary file 2 — Additional file 2: Validation of microarray data for four probe sets by qRT-PCR. In each graph the array data are plotted on the left and qRT-PCR data on the right. Blue bars represent Ofanto data. Red bars represent Cappelli data. CTRL expression data were used as baseline for fold change calculations. The log2 fold change is plotted on the y-axis. The Pearson correlation among microarray FC and qPCR FC is 0.753. ACS: acyl-CoA synthase; ACX: acyl-CoA oxidase; ECH: enoyl-CoA hydratase; HCD: L-β-hydroxyacyl-CoA dehydrogenase; AAT: acyl-CoA-acetyltransferase enzyme or thiolase. (PPTX 130 KB) [file 12864_2013_5521_MOESM2_ESM.pptx]

## Slide 1
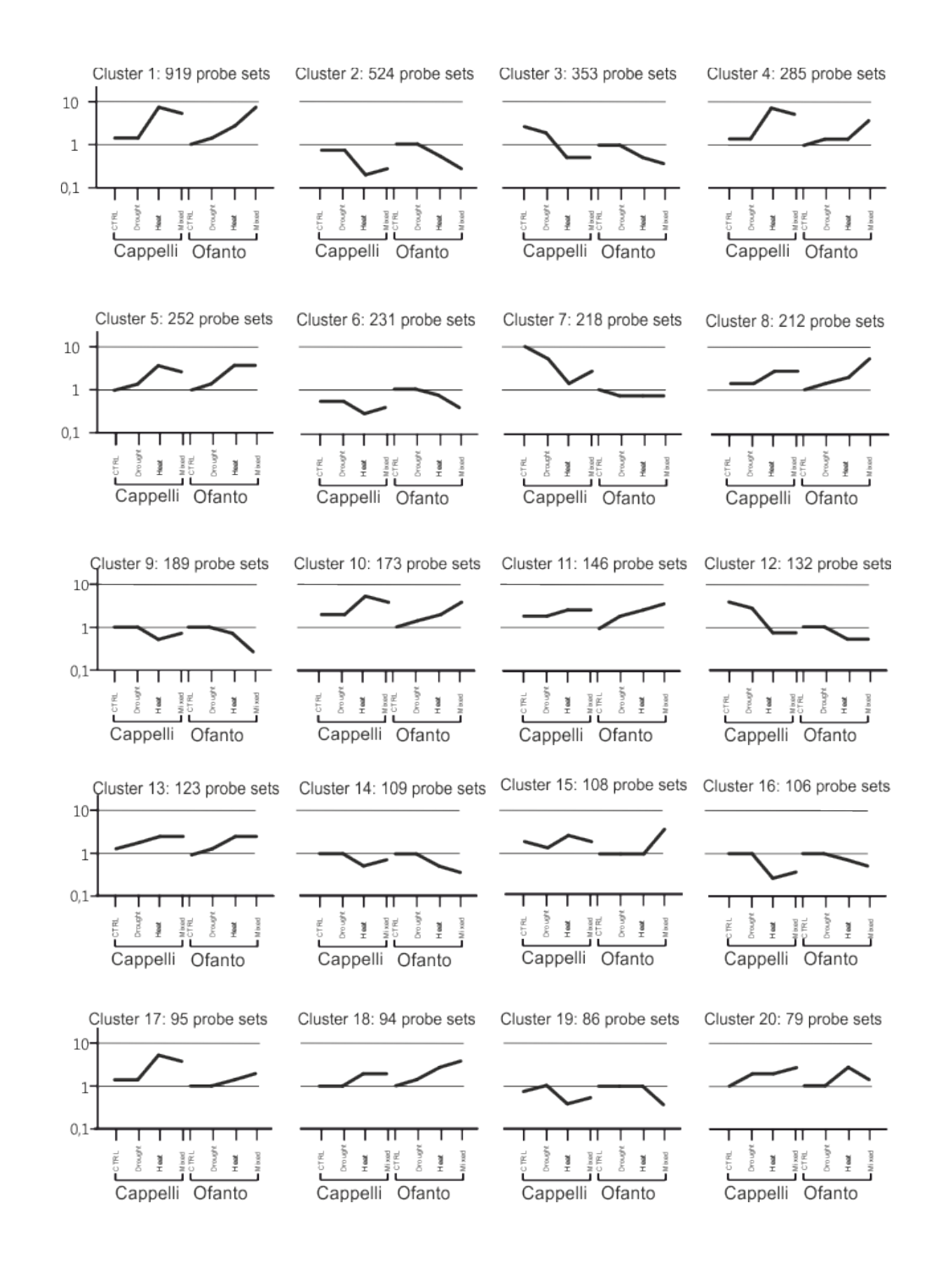

## Slide 2
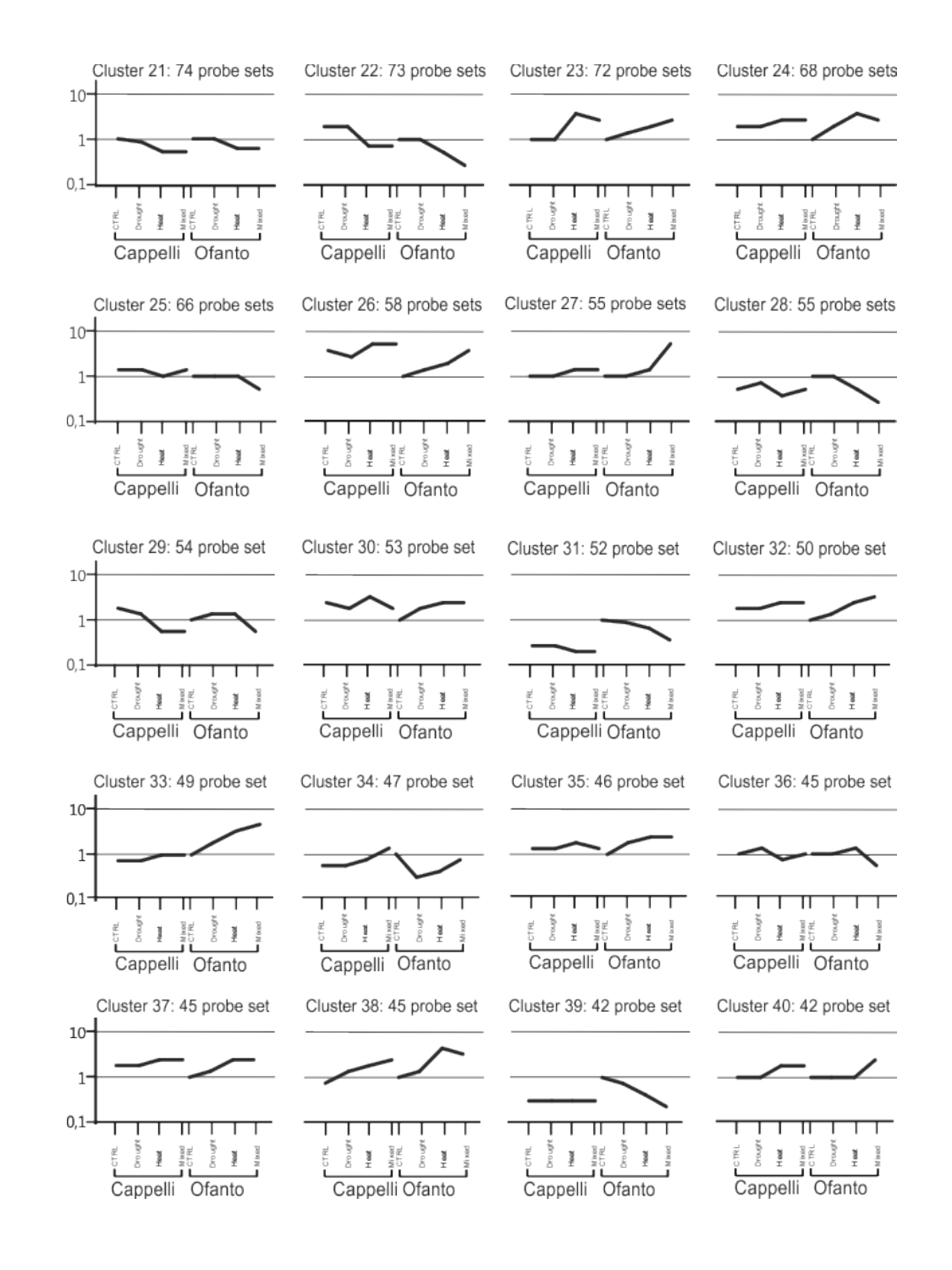

## Slide 3
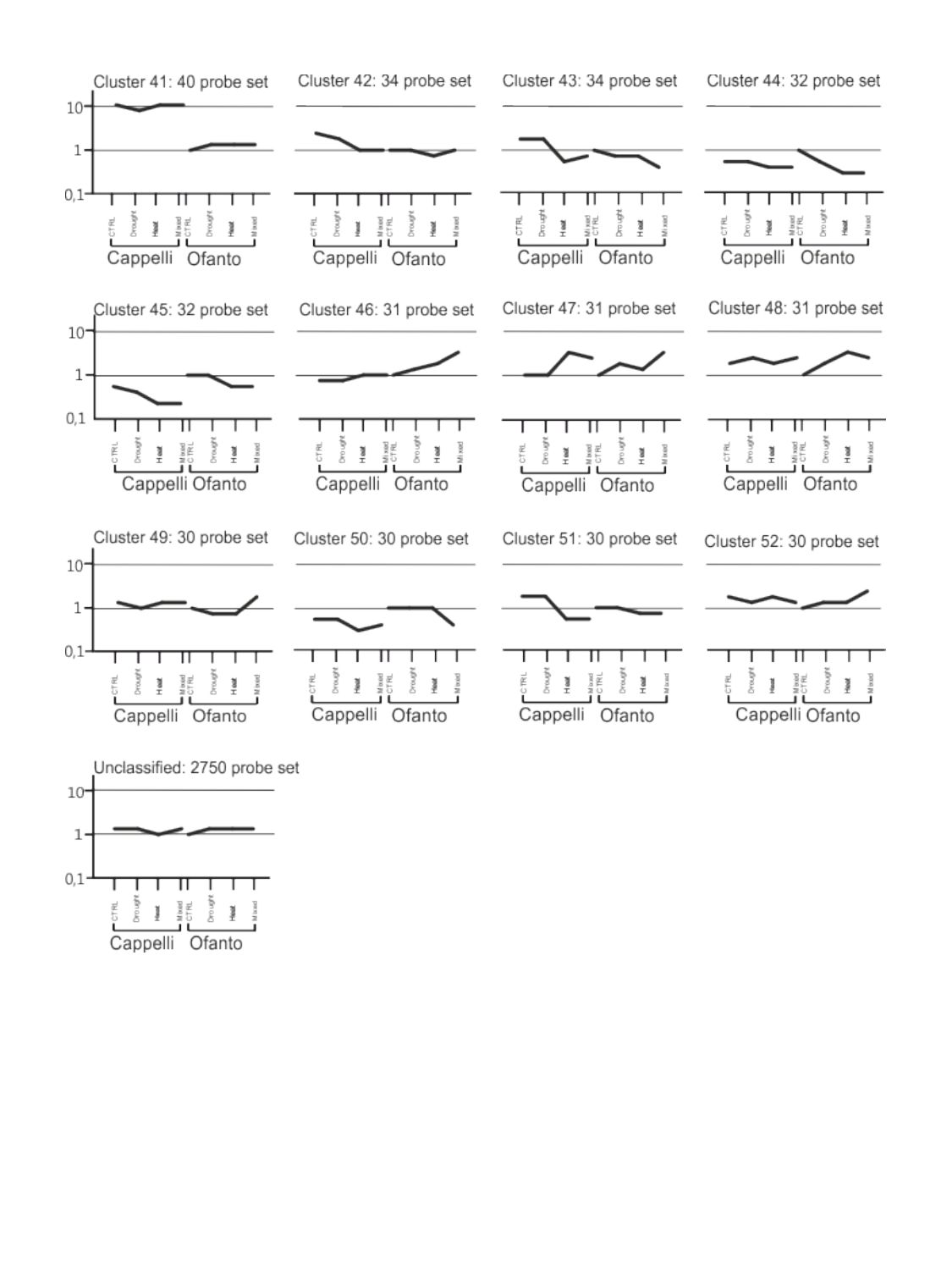

Supplement: Supplementary file 4 — Additional file 4: QT-clustering analysis obtained using the expression values of the 8,660 stress-related genes differentially expressed in at least one condition/genotype. The analyses was performed with a minimum cluster size of 30 and a correlation value of 0.80. The four treatment conditions, grouped by genotypes, are plotted on x axis. The relative expression level (for each probe set the data were normalized to the median expression level of the Ofanto Ctrl samples) is plotted on the y axis. The lines represent the mean expression trend of all probe sets belonging to each cluster. (PPTX 479 KB) [file 12864_2013_5521_MOESM4_ESM.pptx]
