# Supplementary material for: Different stress responsive strategies to drought and heat in two durum wheat cultivars with contrasting water use efficiency
Source: BMC Genomics. 2013 Nov 22;14(1):821. doi: 10.1186/1471-2164-14-821 (PMC4046701; doi:10.1186/1471-2164-14-821)
Supplement: Supplementary file 6 — Additional file 6: Data from Affymetrix hybridization experiments expressed as Fold Change (FC) in black. In comparison with the FCs of microarray data, the FCs of qRT-PCR results from the experiment of young plants at tillering stage subjected to severe drought stress of 27/28% of RSWC are reported in blue. The results showed the differences in gene expression between Ofanto and Cappelli in non stressed/control (Ctrl) vs stressed/drought (Dr) condition in 3rd and 4th columns, while the FC values in the comparisons Ofanto stressed/drought (Dr) vs Cappelli stressed/drought (Dr) and Ofanto non stressed/control (Ctrl) vs Cappelli non stressed/control (Ctrl) are listed in the 5th and 6th columns. (DOCX 24 KB) [file 12864_2013_5521_MOESM6_ESM.docx]

| **Name**  **Probe set ID** | **UNIPROT** | **Of Drought**  vs **Of Ctrl** | **Ca Drought** vs **Ca Ctrl** | **Of Drought** vs **Ca Drought** | **Of Ctrl** vs **Cap Ctrl** |
| --- | --- | --- | --- | --- | --- |
| Ferritin | AAT67051.1 | 3,7 | 0,2 | 9,0 | 0,5 |
| Ta.681.2.S1_a_at | ferritin [Triticum monococcum] | 10,0 | 15,1 | 1,8 | 2,7 |
| 6-SFT | BAB82469.1 | 0,9 | 3,3 | 0,1 | 0,2 |
| Ta.2789.2.S1_at | sucrose:fructan 6-fructosyltransferase [Triticum aestivum] | 346,5 | 19,0 | 8,1 | 0.4 |
| TPA | DAA02101.1 | 6,3 | 1,2 | 2,6 | 0,5 |
| Ta.3145.1.S1_at | TPA: transposase [Oryza sativa] | 7,6 | 4,7 | 1,1 | 0.7 |
| LEA | BAD22766.1 | 5,7 | 1,3 | 3,9 | 0,9 |
| Ta.5913.1.S1_at | LEA protein [Bromus inermis] | 46,3 | 2,3 | 8,3 | 0.4 |
| CRP-TM | BAC41494.1 | 4,6 | 0,5 | 5,2 | 0,5 |
| Ta.13183.1.S1_s_at | cold regulated protein [Triticum aestivum] | 80,3 | 16,7 | 6,8 | 1,4 |
| Cold regulated prot | BAC41494.1 | 4,7 | 0,4 | 6,5 | 0,5 |
| Ta.13183.1.S1_x_at | cold regulated protein [Triticum aestivum] | 118,1 | 27,9 | 7,8 | 1,9 |
| DHN7 13255 | AAF01695.1 | 6,4 | 0,4 | 2,0 | 0,1 |
| Ta.13255.1.S1_at | dehydrin; DHN7 [Hordeum vulgare] | 3725,8 | 467,4 | 3,9 | 0.5 |
| Cp33HV | CAA11894.1 | 1,1 | 3,2 | 0,5 | 1,5 |
| Ta.18434.1.S1_at | cp33Hv [Hordeum vulgare subsp. vulgare] pir\|\|T05730 probable RNA-binding protein cp33 precursor - barley | 1,5 | 1,2 | 1,2 | 0.9 |
| TLP | AAM15877.1 | 7,7 | 0,6 | 0,2 | 0,0 |
| Ta.25053.1.S1_at | thaumatin-like protein [Triticum aestivum] | 4,3 | 3,5 | 1,7 | 1,4 |
| UP | BAD45939.1 | 3,3 | 1,0 | 9,4 | 2,8 |
| Ta.28273.1.S1_x_at | unknown protein [Oryza sativa (japonica cultivar-group)] sp\|Q9LRI7\|OSR8_ORYSA Hydrophobic protein OSR8 dbj\|BAB03289.1\| hydrophobic polypepti | 3,4 | 3,8 | 1,3 | 1,5 |
| PSII | CAA66373.1 | 1,5 | 7,2 | 0,7 | 3,3 |
| Ta.28750.2.A1_x_at | 10kD PSII protein [Hordeum vulgare] pir\|\|T06173 photosystem II 10K protein precursor - barley sp\|Q40070\|PSBR_HORVU Photosystem II 10 kDa pol | 15,7 | 12,7 | 1,1 | 0.9 |
| Putative protein | NP_916744.1 | 16,2 | 1,3 | 3,0 | 0,3 |
| Ta.29464.1.A1_at | P0042A10.26 [Oryza sativa (japonica cultivar-group)] dbj\|BAB90084.1\| P0042A10.26 [Oryza sativa (japonica cultivar-group)] dbj\|BAB21146.1\| P0 | 132,8 | 16,1 | 5,6 | 0.7 |
| RHSF11 | AAQ23065.1 | 4,0 | 1,0 | 16,3 | 3,9 |
| TaAffx.34778.1.S1_at | heat shock factor RHSF11 [Oryza sativa (japonica cultivar-group)] ref\|NP_916438.1\| putative heat shock transcription factor | 2,6 | 6,3 | 2,6 | 1 |
| HEL | Unknown | 5,5 | 1,2 | 39,7 | 8,8 |
| TaAffx.100436.1.S1_at | Unknown | 7E-02 | 4.3E-04 | 299,6 | 1,9 |
| NAD(P)H-quinone | AAQ05272.1 | 0,3 | 4,2 | 0,2 | 2,6 |
| TaAffx.112816.1.S1_at | NADH dehydrogenase subunit B [Cycas revoluta] | 0.6 | 0.5 | 1,5 | 1,2 |
