# Supplementary material for: Different stress responsive strategies to drought and heat in two durum wheat cultivars with contrasting water use efficiency
Source: BMC Genomics. 2013 Nov 22;14(1):821. doi: 10.1186/1471-2164-14-821 (PMC4046701; doi:10.1186/1471-2164-14-821)
Supplement: Supplementary file 8 — Additional file 8: Primer lists used to find expression level polymorphisms and to validate microarray data. (DOCX 19 KB) [file 12864_2013_5521_MOESM8_ESM.docx]

| **Names** | **Probe set ID** | **Primer sequence forward** | **Primer sequence reverse** | **Amplicon length (bp)** |
| --- | --- | --- | --- | --- |
| Ferritin | Ta.681.2.S1_a_at | 5´ TCGACCAGATGCTGCTTGAG 3’ | 5´ CATCCTCCATTGCCCGTAGT 3’ | 69 |
| 6-SFT | Ta.2789.2.S1_at | 5’ CCAAGGAAGGCCAAGACATATG 3’ | 5’ GACCCAAGGATAGATGTTTCAATTC 3’ | 75 |
| TPA | Ta.3145.1.S1_at | 5’ CAATAAGAATGAACTTACGAGTATGCAGTA 3’ | 5’ TTTCGTGGGAAAATCGACAGA 3’ | 71 |
| LEA | Ta.5913.1.S1_at | 5’ ATCACAAAGAGCCGCCACTA 3’ | 5’ TCGGACACACCACAGGAAT 3’ | 100 |
| CRP-TM | Ta.13183.1.S1_s_at | 5’ GATTTTGACACTCGGCTCTGT 3’ | 5’ TCGTTGCCATGCGTATACAGG 3’ | 105 |
| Cold regulated protein | Ta.13183.1.S1_x_at | 5’ TTTTGACACTCGGCTCTGTG 3´ | 5’ GCACGCTACAGAACAGACCA 3´ | 83 |
| DHN7 13255 | Ta.13255.1.S1_at | 5’ GCCACCTCTTGCAGAATAATAAGAT 3’ | 5’ ATTCAAGTCCACAAAACTCAGAACTC 3’ | 81 |
| Cp33HV | Ta.18434.1.S1_at | 5’ GTTCAAAAATTCTGCCGTTCCA 3´ | 5’ CGTGGCTGAAATCCCACTTC 3´ | 80 |
| TLP | Ta.25053.1.S1_at | 5’ CGGCGGCGACACCTATT 3’ | 5’ TTCCCCTTGAAGAACTTCGAGTA 3’ | 81 |
| UP | Ta.28273.1.S1_x_at | 5’ GGAGAGCGGCAGGGACTAC 3’ | 5’ CACGGCATGAGGTGCTACAC 3’ | 58 |
| PSII | Ta.28750.2.A1_x_at | 5’ GCTGTTTCTCTGGGCAATCAC 3’ | 5’ AAGCCAAGGCACTAGTATTGTAGACA 3’ | 80 |
| Putative protein | Ta.29464.1.A1_at | 5’ GTCAATTTTGTTCGGCTCATCTT 3’ | 5’ CGATTTGCATCAACCAACG 3’ | 78 |
| RHSF11 | TaAffx.34778.1.S1_at | 5’ GCCGTTCCCCTTCTCTAATC 3’ | 5’ CATAAACTCCAAAACATCACACG 3’ | 106 |
| HEL | TaAffx.100436.1.S1_at | 5’ GGATTGTCAGGATGGCTATGTG 3’ | 5’ TCAGTAAGACCAACACCATTTTTAGG 3’ | 96 |
| NAD(P)H-quinone | TaAffx.112816.1.S1_at | 5’ CCCCCTAGAGTAGCTGTTAATACGAA 3’ | 5’ GTTCAACTTTATGTATTCCTCTATCCGTAGA 3’ | 100 |
| OEP16-3; | Ta.12727.1.S1_at | 5’ GATGCACCTAGTTCCTTTGCAA 3’ | 5’ GCAAGATATTTATTCCAGGTCTGTTG 3’ | 81 |
| AAT | Ta.28367.1.S1_at | 5’ CAGCGTGCACGAAAGATTTA 3’ | 5’ TAGAGGCGTGAAACCAAACC 3’ | 164 |
| ACL | TaAffx.31738.1.S1_at | 5’ GTGGAGAAGGTGGTGTACATGC 3’ | 5’ AAAACACAGCGGAACACAGAAG 3’ | 187 |
| ACX | Ta.9335.1.S1_at | 5’ TCCAGCTCCGACTTTGTTCT 3’ | 5’ ACCTGCTGCAGTAGCGAAAT 3’ | 172 |
| ECH/HCD | Ta.9184.1.S1_at | 5’ CTGAACCTCACATGCGTGAC 3’ | 5’ GATGGGCTTTCCCAAGTACA 3’ | 215 |
