# Supplementary material for: Different stress responsive strategies to drought and heat in two durum wheat cultivars with contrasting water use efficiency
Source: BMC Genomics. 2013 Nov 22;14(1):821. doi: 10.1186/1471-2164-14-821 (PMC4046701; doi:10.1186/1471-2164-14-821)
Supplement: Supplementary file 9 — Additional file 9: Nucleotide sequence (223 bp) of HEL gene obtained from cDNA of Ofanto and Cappelli by sequencing analysis in the first part of the table, and in the second part Contig444272 sequence (1448 bp) of Chinese spring obtained from cerealsDB database. (DOCX 17 KB) [file 12864_2013_5521_MOESM9_ESM.docx]

| **GENE** | **Nucleotide sequence** |
| --- | --- |
| **HEL** | GCAACGGCAGAGGTCATTATACTGACCAAGTCAGGGAGTCAAAGGTGATGGATT GTCAGGATGGCTATGTGGAGAGATGCATACGCATCGGTATTACCGGCATTATCTGC CGTTGCTTTCCTAAAAATGGTGTTGGTCTTACTGACCAAGCAATGTTGGCGACGGC TGCCTCACCGAGTAAGATTGACGAGGAGAGACCGTCACCGAAAAACTCCCACGACCA |
|  |  |
|  | **Sequences of HEL gene obtained from sequencing analyses, about 220 bp** |
|  |  |
| **Contig 444272** | caggggtcctaattggtaagaatatctaattaattggctgcttgtgaaggagatgaagtaattaatc atgaacatgtatgaccttaggtgattatcctaatcaagtcagggagtcaaaggtgatggattgtcagg atggctatgtggagagatgcatacagcatcggtactaccggcgttatctgcggttgctttcctaaaaat ggtgttggtcttactgaccaagcaatgttggcgacggctgcctcaccgagtaagatcgacgaggagag accgtcaccgaaaaactcccacgaccaaggtgcatcattggaacacatgccactattgttaggcag gggtcctaattggtaagaatatctaattaattggttgccctgtgaaggagatgaagtaattaatcatga gcacgtatgacctcaggtgattatactgatcaagtcagggagtcaaaggtgatggattgtcgggatgg ctatgtggagagatgcttcgccatcggtacagctgttgtttgcagtttctttcctaaacatggcgtcggtc ttactgatcaagcattgttgtctacatctggcttaccgaggaagatcgacgaggaaatcgctttgccgt agaactcacatgaccaaggtgcgtgattgaaatgcatgccatgtaattagtaagatcttttaattaac tgttgtgtcaaggaacaccccattgcgattcagtatcccagattggggtccttcctacagagtcaaggg acttgtttgtaatttcccatttatgtccaactgtaatcactgtttgtaatatggttatatgaataaagtttg gatccttcgggatccctctctctgttccaaataaggtaggtgatcaacgttcttggcagcaaatataca tgtccgaatggtcaggtattgaaaaatgagtccatcaaatgttccaaagtcctaagtcgtggctatatt cgtgagcgcatgcgctatatacatgcgtggtgtgcatgtagcatagtatgtcccttgcctaccacctca tccaagtggtcaagtgagctttccttgtatgttggtgtagagcattgtccatgggtgaggtgggactaaa cccacatgcactccacccactcatctactctttgtattttccgcaattctgtctggtggccaaggtgtttg cccaactagctattacccctggcccaaaatgctatccatatggatatatcactaccgcaggatgctgc taacgcgacactataatcagagacctttcgacgaaactatgtgcgacgcattaatcgcaaacggtga tgtaaaaaacctgtacaaaaagatgcaaaacgtttgtgatggcggataaatcaaacacagttcata ttataattacgtgtgcgatgagtggcatacggttatccataagaactatttgcgatgagacagaacaa cagaaac |
