# Supplementary material for: Different stress responsive strategies to drought and heat in two durum wheat cultivars with contrasting water use efficiency
Source: BMC Genomics. 2013 Nov 22;14(1):821. doi: 10.1186/1471-2164-14-821 (PMC4046701; doi:10.1186/1471-2164-14-821)
Supplement: Supplementary file 7 [file 12864_2013_5521_MOESM7_ESM.docx]

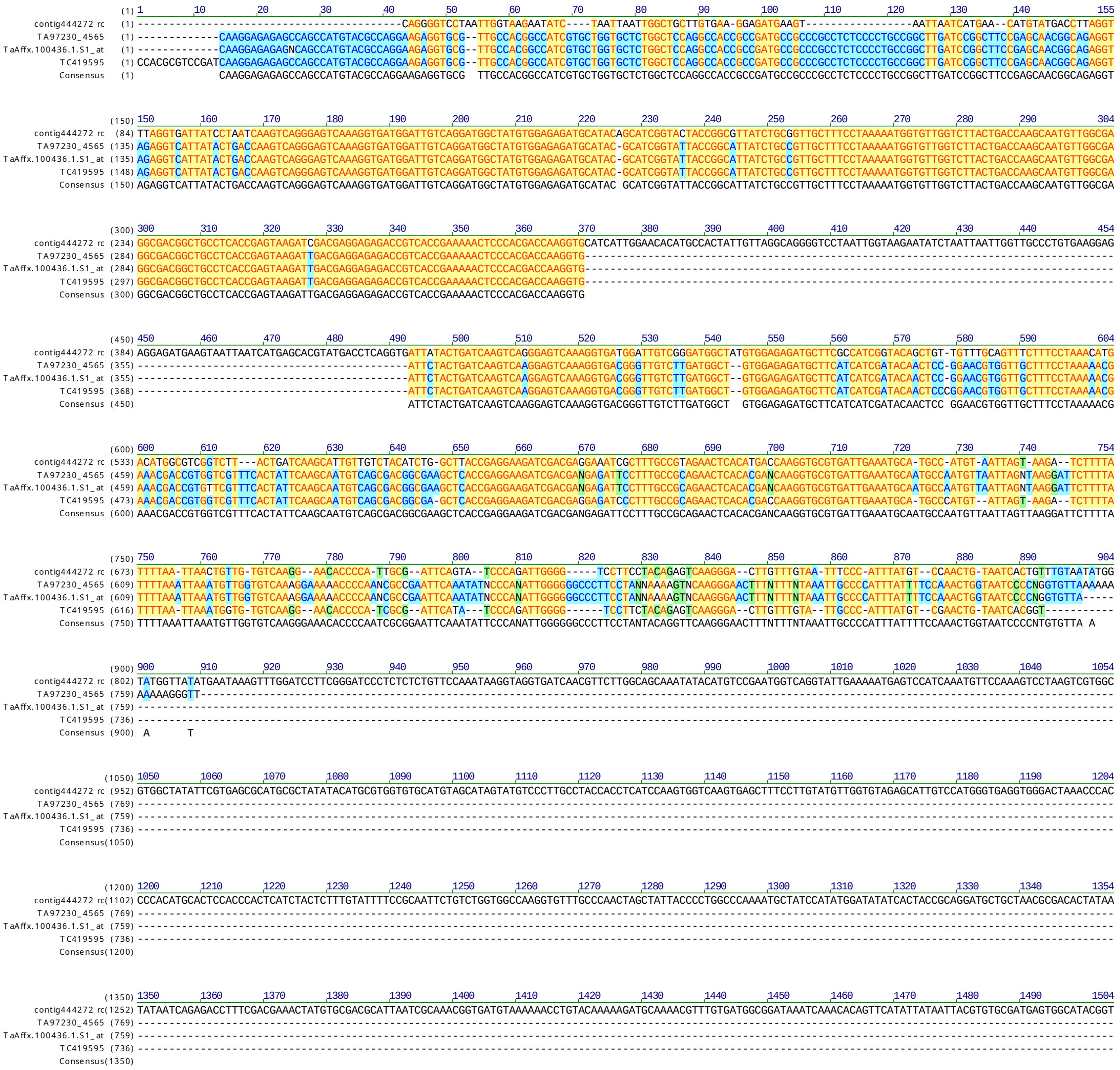
contig444272 rc
TA97230_4565
TaAffx.100436.1.S1_at
TC419595
Consensus
